# A singular value decomposition Bayesian multiple-trait and multiple-environment genomic model

**DOI:** 10.1038/s41437-018-0109-7

**Published:** 2018-08-17

**Authors:** Osval A. Montesinos-López, Abelardo Montesinos-López, José Crossa, Kismiantini, Juan Manuel Ramírez-Alcaraz, Ravi Singh, S. Mondal, P. Juliana

**Affiliations:** 1 Facultad de Telemática, Universidad de Colima, 28040 Colima, Mexico; 2 Departamento de Matemáticas, Centro Universitario de Ciencias Exactas e Ingenierías (CUCEI), Universidad de Guadalajara, 44430 Guadalajara, Jalisco Mexico; 3 International Maize and Wheat Improvement Center (CIMMYT), Apdo. Postal 6-641, 06600 Ciudad de México, Mexico; 4 Department of Mathematics, Yogyakarta State University, Yogyakarta, Indonesia

**Keywords:** Genome evolution, Tropical ecology, Agriculture

## Abstract

Today, breeders perform genomic-assisted breeding to improve more than one trait. However, frequently there are several traits under study at one time, and the implementation of current genomic multiple-trait and multiple-environment models is challenging. Consequently, we propose a four-stage analysis for multiple-trait data in this paper. In the first stage, we perform singular value decomposition (SVD) on the resulting matrix of trait responses; in the second stage, we perform multiple trait analysis on transformed responses. In stages three and four, we collect and transform the traits back to their original state and obtain the parameter estimates and the predictions on these scale variables prior to transformation. The results of the proposed method are compared, in terms of parameter estimation and prediction accuracy, with the results of the Bayesian multiple-trait and multiple-environment model (BMTME) previously described in the literature. We found that the proposed method based on SVD produced similar results, in terms of parameter estimation and prediction accuracy, to those obtained with the BMTME model. Moreover, the proposed multiple-trait method is atractive because it can be implemented using current single-trait genomic prediction software, which yields a more efficient algorithm in terms of computation.

## Introduction

Breeders often want to improve more than one trait simultaneously in their breeding programs and thus conduct various experiments. For example, Teixeira et al. (2016) reported that a breeding program in Brazil measured 41 pig traits obtained by crossing 345 F2 pig populations of Brazilian Piau × commercial pigs. To analyze this type of experiment, breeders implement one of the following two approaches: (i) they perform a univariate analysis (one trait at a time), therefore ignoring the correlation between traits that does not allow to improve either parameter estimates or prediction accuracy, or (ii) they perform a multiple-trait analysis, which may not only take into account the correlation between traits but may also significantly increase the computing intensity.

Implementing first approach is valid when the correlation between traits is low or close to zero, but it is less desirable when the correlation between traits is moderate to strong. However, implementing the second approach is sometimes challenging—for example, when there are a large number of traits, and under these circumstances, breeders opt for the first approach. Furthermore, implementing the second approach is also challenging because early breeding programs start with at least 1000 lines that are evaluated in multiple environments, which complicates the analysis, as including genotype × environment (G × E) increases the dimensionality of the data considerably (Chiquet et al. 2013).

As mentioned above, multiple-trait analysis improves parameter estimates and prediction accuracy. With regard to parameter estimates, Schulthess et al. (2017) found that multiple-trait analysis improves parameter estimates, while Calus and Veerkamp (2011) found modest improvement using multiple-trait analysis compared to separate-trait analysis for prediction accuracy; these authors also showed that the performance of multiple-trait analysis depends considerably on whether only some traits are missing in only some individuals or in all individuals. Jia and Jannink (2012) also showed evidence favoring multiple-trait analysis compared to single-trait analysis, finding that the genetic correlation between traits is the basis for the benefit of multiple-trait analysis. Jiang et al. (2015) later arrived at the same conclusion in favor of multiple-trait analysis. Montesinos-López et al. (2016) also found modest improvement in the prediction accuracy of multiple-trait analysis when comparing correlated traits to the analysis that assumes null correlation between traits. Along these lines, He et al. (2016) concluded that modeling multiple traits could improve the prediction accuracy for correlated traits in comparison to univariate-trait analysis. Schulthess et al. (2017) also found that multiple-trait analysis is better in terms of prediction accuracy than separate-trait analysis, pointing out that the multiple-trait model is better when the degree of relatedness between genotypes is weaker.

There is evidence suggesting that even when traits are correlated, genomic-enabled prediction accuracy is not improved, as stated by Montesinos-López et al. (2017a) and as shown by Märtens et al. (2016), who compared multiple-trait analysis to single-trait analysis and found no difference in terms of prediction accuracy. This issue was also documented by Oliveira and Teixeira-Pinto (2015) who proved this result by stating that, in the multivariate linear regression case (when the covariates in each equation are the same), even if the errors are strongly correlated, the multivariate model gives the same result (both point estimates and standard errors) as fitting individual regressions with ordinal least squares for each outcome, despite the level of correlation between the errors.

As a breeding tool, genomic selection (GS) uses all available molecular markers (Meuwissen et al. 2001) to design genomic-assisted breeding programs and develop new marker-based models for genetic evaluation. GS provides opportunities to obtain higher rates of genetic gain than traditional phenotypic selection in less time and at a reasonable cost. For example, in animal breeding, GS allows animal scientists to select young animals early in life that do not have records, greatly reducing evaluation costs and generation intervals when compared to the traditional progeny test schemes (Schaeffer 2006; Boichard et al. 2016). In general, for traits that have a long generation time or are difficult to evaluate (i.e., insect resistance, bread-making quality, and others), GS is cheaper and/or easier than traditional phenotypic selection because more candidates can be characterized for a given cost, thus enabling increased selection intensity. Hence, GS has a number of merits over traditional selection because it reduces selection duration and increases selection accuracy, intensity, efficiency, and gains per unit of time. In addition, it saves time and financial investment, along with producing reliable results (Rutkoski et al. 2011; Desta and Ortiz 2014). This enables faster development of improved crop varieties to cope with the challenges of climate change and the decrease in arable land (Bhat et al. 2016).

Therefore, we propose an alternative method for analyzing multiple-trait and multi-environment data—one that takes into account the correlation between traits. This model will be useful for analyzing multiple-trait and multi-environment data because the linear predictor may include the following interaction terms: environment × trait, genotype × trait, and three-way interaction (environment × genotype × trait), assuming an unstructured variance–covariance matrix in the genetic and residual covariance matrices of traits and an identity matrix for the correlation matrix between environments.

The proposed method consists of four steps. In the first step, we transform the original matrix of response variables into a matrix of response variables of the same dimension but between uncorrelated transformed traits using singular value decomposition (SVD), which is equivalent to using principal component analyses (PCA). This first step is performed ignoring all the information of the design effect and other covariates. In the second step, given that the traits are not correlated, we apply a single-trait analysis where we can take into account the design effect, along with the effects of genotypes, environments, genotype × environment interaction, and other covariates, if they are available. In the third step, we collect and put together all the parameter estimates of the single analysis, which are transformed in the fourth step to obtain the parameter estimates and/or predictions for the traits in the original scale of the multiple-trait and multiple-environment data. Our approach has the same goal as the canonical transformation method proposed by Thompson (1977), which involves using special matrices to transform the observations on several correlated traits into new variables that are uncorrelated to each other, which means that these new variables can be analyzed as single-trait analysis, but the results (predictions) are transformed back to the original scale of the observations (Mrode 2014). However, our method is different to the Thompson (1977) method since our approach directly decorrelates the matrix of response variables with the SVD, while the Thompson (1977) method transforms the variance of the response var(***Y***) = **Σ**_***t***_ + ***R***, such that ***QRQ***^***T***^ = ***I*** and ***Q*Σ**_***t***_***Q***^***T***^ = ***W***, where ***I*** is an identity matrix and ***W*** is a diagonal matrix, of course assuming that **Σ**_***t***_ and ***R*** are positive definite matrices and that there is a matrix ***Q***. More details of this method can be found in chapter 6 of the book by Mrode (2014). A detailed example of the Thompson (1977) method is available in Appendix E, section E.1, in the book by Mrode (2014). The Thompson (1977) method has the inconvenience that if the (co)variances ***R*** and **Σ**_***t***_ are unknown, the transformations mentioned above need to be applied at each iteration of the Henderson mixed model equations. For this reason, it's implementation is not straightforward using current univariate software.

The advantage of the proposed alternative method is that the analysis can be performed directly using the current software for univariate genomic selection and prediction. However, it is important to point out that when the distribution of the traits has considerably departed from normality, the proposed method does not guarantee independence between the transformed traits, a key assumption for the successful implementation of the proposed model, since for non-normal traits lack of correlation does not imply independence. Also, the proposed method can be implemented to perform the analysis even when there are some missing traits per individual and some individuals are missing in some environments, as long as the level of unbalance is not strong.

## Materials and methods

### Statistical models

#### Multiple-trait multiple-environment model

Since genotype × environment interaction is of paramount importance in plant breeding, the following univariate linear mixed model is usually used for each trait:

1 $$y_{ij} = E_i + g_j + gE_{ij} + e_{ij}$$

where *y*_*ij*_ represents the normal response from the *j*th line in the *i*th environment (*i* = 1, 2, …, *I*, *j* = 1, 2, …, *J*). *E*_*i*_ represents the effect of the *i*th environment and is assumed as a fixed effect, *g*_*j*_ represents the random effect of the genomic effect of the *j*th line, with ***g*** = (*g*_*j*_, …, *g*_*J*_)^*T*^ ~ $N\left( {{\mathbf{0}},\sigma _1^2{\kern 1pt} {\boldsymbol{G}}_g} \right)$, $\sigma _1^2$ denotes the genomic variance and ***G***_*g*_ is of order *J* × *J* and represents the genomic relationship matrix (GRM) and is calculated using the Van Raden (2008) method as ***G***_*g*_ = $\frac{{{\boldsymbol{ZZ}}^T}}{p}$, where *p* denotes the number of markers and **Z** the matrix of markers of order *J* × *p*. The ***G***_***g***_ covariance matrix is constructed using the observed similarity at the genomic level between lines, rather than the expected similarity based on pedigree. *gE*_*ij*_ is the random interaction term between the genomic effect of the *j*th line and the *i*th environment where ***gE*** = (*gE*_11_, …, *gE*_*IJ*_)^*T*^ ~ $N\left( {{\mathbf{0}},\sigma _2^2{\kern 1pt} {\bf{I}}_I \otimes {\boldsymbol{G}}} \right)$, $\sigma _2^2$ denotes the variance of the interaction term of genotype by environment, and *e*_*ij*_ is a random error term associated with the *j*th line in the *i*th environment distributed as *N*(0, *σ*^2^), with *σ*^2^ denoting the residual variance. This model is usually used for each of the *l* = 1, …, *L* traits, where *L* denotes the number of traits under study. Next we will present the multivariate version of model (1); for this reason, first we provide the notation for the matrix variate normal distribution, which is a generalization of the multivariate normal distribution. In particular, let the (*n* × *p*) random matrix, ***M***, be distributed as matrix variate normal distribution denoted as ***M*** ~ NM_*n***×***p*_(**Η**, **Ω**, **Σ**), if and only if, the (*np* × 1) random vector vec(***M***) is distributed as multivariate normal denoted as *N*_*np*_(vec(**Η**), **Σ** ⊗ **Ω**); therefore, NM_*n*×*p*_ denotes the (*n* × *p*) dimensional matrix variate normal distribution, **Η** is a (*n* × *p*) location matrix, **Σ** is a (*p* × *p*) first covariance matrix, and **Ω** is a (*n* × *n*) second covariance matrix (Srivastava and Khatri 1979). vec(.) and ⊗ are the standard vector operator and Kronecker product, respectively.

To account for the correlation between traits, all of the *L* traits given in Eq. (1) are jointly modeled in a whole multiple-trait, multiple-environment mixed model as follows:

2 $${\boldsymbol{Y}} = {\boldsymbol{X\beta }} + {\boldsymbol{Z}}_1{\boldsymbol{b}}_1 + {\boldsymbol{Z}}_2{\boldsymbol{b}}_2 + {\boldsymbol{e}}$$

where ***Y*** is of order *n* × *L*, ***X*** is of order *n* × *I*, ***β*** is of order *I* × *L*, ***Z***_1_ is of order *n* × *J*, ***b***_1_ is of order *J* × *L* and contains the first interaction term genotype × trait, ***Z***_2_ is of order *n* × *IJ*, ***b***_2_ is of order *IJ* × *L* and contains the second interaction term genotype × environment × trait, and ***e*** is of order *n* × *L*, with ***b***_1_ distributed under matrix variate normal distribution as NM_*J*×*L*_ (**0**, ***G***_*g*_, **Σ**_*t*_), where **Σ**_*t*_ is the unstructured genetic (co)variance matrix of traits of order *L* × *L*, ***b***_2_ ~ NM_*JI*×*L*_(**0**, **Σ**_*E*_ ⊗ ***G***_***g***_, **Σ**_*t*_), where **Σ**_*E*_ is an unstructured (co)variance matrix of order *I* × *I*, and ***e*** ~ NM_*n*×*L*_(**0**, ***I***_*n*_, ***R***_*e*_), where ***R***_*e*_ is the unstructured residual (co)variance matrix of traits of order *L* × *L*, and ***G***_*g*_ is the GRM described above. The Bayesian multiple-trait and multiple-environment (BMTME) model resulting from Eq. (2) was implemented by Montesinos-López et al. (2016).

First, we provided a modified version of the original BMTME model proposed by Montesinos-López et al. (2016), and in the next section, we will provide the modified Gibbs sampler for this modified BMTME model.

### Gibbs sampler for the BMTME model

Outlined below is the Gibbs sampler for estimating the parameter of interest in the BMTME model. While the order is somewhat arbitrary, we suggest the following:

Step 1. Simulate ***β*** according to the normal distribution given in Supplementary material (A.1).

Step 2. Simulate ***b***_1_ according to the normal distribution given in Supplementary material (A.2).

Step 3. Simulate ***b***_2_ according to the normal distribution given in Supplementary material (A.3).

Step 4. Simulate **Σ**_*t*_ according to the inverse Wishart (IW) distribution given in Supplementary material (A.4).

Step 5. Simulate **Σ**_*E*_ according to the IW distribution given in Supplementary material A (A.5).

Step 6. Simulate ***R***_*e*_ according to the IW distribution given in Supplementary material A (A.6).

Step 7. Return to step 1 or terminate when chain length is adequate to meet convergence diagnostics.

The main differences between this Gibbs sampler and that given by Montesinos-López et al. (2016) are: (i) that this modified Gibbs sampler assumes an unstructured variance–covariance matrix for environments, while the original BMTME model assumes a diagonal variance–covariance matrix for environments, and (ii) that the original BMTME model used non-informative priors based on the Half-*t* distribution of each standard deviation term and uniform priors on each correlation of the covariance matrices of traits (genetic and residual). The modified BMTME model presented here assumes weak informative priors not based on the Half-*t* distribution of each standard deviation (details of the hyperparameters of the BMTME model are given in Supplementary material). The hyperparameters for the BMTME model were set similar to those used in the BGLR software (Pérez-Rodríguez and de los Campos 2014). The modified full conditional of this modified BMTME model, which supports the Gibbs sampler given above, is provided in Supplementary material. Next, we will describe the parameterization of the model given in Eq. (2) to develop the proposed alternative method that is based on SVD.

### BMTME_Thompson version

Following Thompson (1977) and Ducrocq and Chapuis (1993), we define ***Q*** as a matrix of order *L* × *L* such that ***Q*Σ**_*t*_***Q***^*T*^ = ***D***_*t*_, where ***D***_*t*_ is a diagonal matrix also of order *L* × *L* and ***QR***_*e*_***Q***^*T*^ = ***I***_*t*_. The ***Q*** matrix always exists and can be calculated as ***Q*** = ***L***^*T*^***P***, where ${\boldsymbol{P}} = {\bf{U}}_e{\boldsymbol{B}}_e^{ - 0.5}{\boldsymbol{U}}_e^T$, where **U**_*e*_ and **B**_*e*_ are obtained by applying the SVD to ${\bf{R}}_e = {\bf{U}}_e{\bf{B}}_e{\boldsymbol{U}}_e^T$, while ***L***^*T*^ is obtained also by applying the SVD to ***P*Σ**_*t*_***P***^*T*^ = ***LD***_*t*_***L***^*T*^. Then, by applying a linear transformation to Eq. (2), we obtain:

3 $$\begin{array}{*{20}{l}} {{\it{\boldsymbol{Y}}}{\boldsymbol{Q}}^T} \hfill & = \hfill & {{\boldsymbol{X\beta Q}}^T + {\boldsymbol{Z}}_1{\boldsymbol{b}}_1{\boldsymbol{Q}}^T + {\boldsymbol{Z}}_2{\boldsymbol{b}}_2{\boldsymbol{Q}}^T + {\boldsymbol{eQ}}^T} \hfill \cr {} \hfill & {\boldsymbol{Y}^\& } = \hfill & {{\boldsymbol{X\beta }}^\& + {\boldsymbol{Z}}_1{\boldsymbol{b}}_1^\& + {\boldsymbol{Z}}_2{\boldsymbol{b}}_2^\& + {\boldsymbol{Qe}}^\& } \hfill \end{array}$$

Then note that:

$$\begin{array}{*{20}{l}} {\mathrm{Var}\left( {\mathrm{vec}\left( {{\boldsymbol{b}}_1^\& } \right)} \right)} \hfill & = \hfill & {\left( {{\boldsymbol{Q}} \otimes {\boldsymbol{I}}_J} \right)\mathrm{Var}\left( {\mathrm{vec}\left( {{\boldsymbol{b}}_1} \right)} \right)\left( {{\boldsymbol{Q}}^T \otimes {\boldsymbol{I}}_J} \right)} \hfill \cr {} \hfill & = \hfill & {{\boldsymbol{Q}}{\mathbf{\Sigma }}_t{\boldsymbol{Q}}^T \otimes {\mathbf{G}}_g = {\mathbf{D}}_t \otimes {\mathbf{G}}_g} \hfill \cr {\mathrm{Var}\left( {\mathrm{vec}\left( {{\boldsymbol{b}}_2^\& } \right)} \right)} \hfill & = \hfill & {\left( {{\boldsymbol{Q}} \otimes {\boldsymbol{I}}_{JI}} \right)\mathrm{Var}\left( {\mathrm{vec}\left( {{\boldsymbol{b}}_2} \right)} \right)\left( {{\boldsymbol{Q}}^T \otimes {\boldsymbol{I}}_{JI}} \right)} \hfill \cr {} \hfill & = \hfill & {{\boldsymbol{Q}}{\mathbf{\Sigma }}_t{\boldsymbol{Q}}^T \otimes {\mathbf{\Sigma }}_E \otimes {\mathbf{G}}_g = } \hfill \cr {} \hfill & = \hfill & {{\mathbf{D}}_t \otimes {\mathbf{\Sigma }}_E \otimes {\mathbf{G}}_g} \hfill \cr {\mathrm{Var}\left( {\mathrm{vec}\left( {{\boldsymbol{e}}^\& } \right)} \right)} \hfill & = \hfill & {\left( {{\boldsymbol{Q}} \otimes {\boldsymbol{I}}_n} \right)\mathrm{Var}\left( {\mathrm{vec}\left( {\boldsymbol{e}} \right)} \right)\left( {{\boldsymbol{Q}}^T \otimes {\boldsymbol{I}}_n} \right)} \hfill \cr {} \hfill & = \hfill & {{\boldsymbol{Q}}{\mathbf{R}}_e{\boldsymbol{Q}}^T \otimes {\mathbf{I}}_n = {\mathbf{I}}_t \otimes {\mathbf{I}}_n} \hfill \end{array}$$

Since **D**_*t*_ and **I**_*t*_ are diagonal matrices of order *t* × *t*, the full conditional distributions for the transformed random effects ${\boldsymbol{b}}_1^\&$ and ${\boldsymbol{b}}_2^\&$ of the BMTME model are:

Full conditional for $\mathrm{vec}\left( {{\boldsymbol{b}}_1^\& } \right)$

4 $$P\left( {\mathrm{vec}\left( {{\boldsymbol{b}}_1^\& } \right)|\mathrm{ELSE}} \right) \propto N\left( {\mathrm{vec}\left( {{\tilde{\boldsymbol b}}_1^\& } \right),\widetilde {\bf{\Sigma }}_{{\boldsymbol{b}}_1^\& }} \right)$$

where $$\widetilde {\bf{\Sigma }}_{{\boldsymbol{b}}_1^\& }$$ = $$( {{\bf{D}}_t^{ - 1} \otimes {\boldsymbol{G}}_g^{ - 1} + {\boldsymbol{I}}_L \otimes {\boldsymbol{Z}}_1^T{\boldsymbol{Z}}_1} )^{ - 1}$$ and $$\mathrm{vec}( {\widetilde {\boldsymbol{b}}_1^\& } )$$ = $$\widetilde {\bf{\Sigma }}_{{\boldsymbol{b}}_1^\& }\left( {{\boldsymbol{I}}_L \otimes {\boldsymbol{Z}}_1^T} \right)\left[ {vec\left( {\boldsymbol{Y}^\& } \right) - vec\left( {{\boldsymbol{X\beta }}^\& } \right) - vec({\boldsymbol{Z}}_2{\boldsymbol{b}}_2^\& )} \right]$$.

Full conditional for $vec\left( {{\boldsymbol{b}}_2^\& } \right)$

5 $$P\left( {vec\left( {{\boldsymbol{b}}_2^\& } \right)|ELSE} \right) \propto N\left( {vec\left( {\widetilde {\boldsymbol{b}}_2^\& } \right),\widetilde {\bf{\Sigma }}_{{\boldsymbol{b}}_2^\& }} \right)$$

where $$\widetilde {\bf{\Sigma }}_{{\boldsymbol{b}}_2^\& }$$ = $$( {{\bf{D}}_t^{ - 1} \otimes {\mathbf{\Sigma }}_E^{ - 1} \otimes {\boldsymbol{G}}_g^{ - 1} + {\boldsymbol{I}}_L \otimes {\boldsymbol{Z}}_2^T{\boldsymbol{Z}}_2} )^{ - 1}$$ and $$vec\left( {\widetilde {\boldsymbol{b}}_2^\& } \right)$$ = $$\widetilde {\bf{\Sigma }}_{{\boldsymbol{b}}_2^\& }\left( {{\boldsymbol{I}}_L \otimes {\boldsymbol{Z}}_2^T} \right)\left\{ {\mathrm{vec}\left( {\boldsymbol{Y}^\& } \right) - \mathrm{vec}\left( {{\boldsymbol{X\beta }}^\& } \right) - \mathrm{vec}\left( {{\boldsymbol{Z}}_1{\boldsymbol{b}}_1^\& } \right)} \right\}$$.

It is important to point out that the full conditionals of ${\boldsymbol{b}}_1^\&$ and ${\boldsymbol{b}}_2^\&$ are diagonals for traits; for this reason, these full conditionals can be sampled independently for each trait, as:

Full conditional for ${\boldsymbol{b}}_1^{\& (l)}$ for *l* = 1, 2, …, *L*

6 $$P\left( {{\boldsymbol{b}}_1^{\& (l)}|\mathrm{ELSE}} \right) \propto N\left( {\widetilde {\boldsymbol{b}}_1^{\& (l)},\widetilde {\bf{\Sigma }}_{{\boldsymbol{b}}_1^{\& (l)}}} \right)$$

where $$\widetilde {\bf{\Sigma }}_{{\boldsymbol{b}}_1^{\& ({\boldsymbol{l}})}}$$ = $$\left( {d_l^{ - 1} \otimes {\boldsymbol{G}}_g^{ - 1} + {\boldsymbol{Z}}_1^T{\boldsymbol{Z}}_1} \right)^{ - 1}$$ and $$\widetilde{\boldsymbol{b}}_1^{\& (l)} = \widetilde{\bf{\Sigma }}_{{\boldsymbol{b}}_1^\& }( {{\boldsymbol{Z}}_1^T} )[ {{\boldsymbol{Y}}^{\& (l)} - {\boldsymbol{X\beta }}^{\& (l)} - {\boldsymbol{Z}}_2{\boldsymbol{b}}_2^{\& (l)}} ]$$.

Full conditional for ${\boldsymbol{b}}_2^{\& (l)}$ for *l* = 1, 2, …, *L*

7 $$P\left( {{\boldsymbol{b}}_2^{\& (l)}|\mathrm{ELSE}} \right) \propto N\left( {\widetilde {\boldsymbol{b}}_2^{\& (l)},\widetilde {\bf{\Sigma }}_{{\boldsymbol{b}}_2^{\& (l)}}} \right)$$

where $$\widetilde {\bf{\Sigma }}_{{\boldsymbol{b}}_2^{\& (l)}}$$ = $$( {d_l^{ - 1} \otimes {\mathbf{\Sigma }}_E^{ - 1} \otimes {\boldsymbol{G}}_g^{ - 1} + {\boldsymbol{Z}}_2^T{\boldsymbol{Z}}_2} )^{ - 1}$$ and $$\widetilde {\boldsymbol{b}}_2^{\& (l)}$$ = $$\widetilde {\bf{\Sigma }}_{{\boldsymbol{b}}_2^{\& ({\boldsymbol{l}})}}\left( {{\boldsymbol{Z}}_2^T} \right)\left\{ {\boldsymbol{Y}^{\& (l)} - {\boldsymbol{X\beta }}^{\& (l)} - {\boldsymbol{Z}}_1{\boldsymbol{b}}_1^{\& (l)}} \right\}$$.

Therefore, the Gibbs sampler for the BMTME Thompson version should be:

Step 1. Simulate ***β*** according to the normal distribution given in Supplementary material (A.1).

Step 2. Simulate ${\boldsymbol{b}}_1^{\& (l)}$ for *l* = 1, 2, …, *L* according to the normal distribution given in Eq. (6).

Step 3. Simulate ${\boldsymbol{b}}_2^{\& (l)}$ for *l* = 1, 2, …, *L* according to the normal distribution given in Eq. (7).

Step 4. Then transform back ***Y*** = ***Y***^&^***Q***^(−1)*T*^, ${\boldsymbol{\beta }} = {\boldsymbol{\beta }}^\& {\boldsymbol{Q}}^{( - 1)T}$, ${\boldsymbol{b}}_1 = {\boldsymbol{b}}_1^\& {\boldsymbol{Q}}^{( - 1)T}$ and ${\boldsymbol{b}}_2 = {\boldsymbol{b}}_2^\& {\boldsymbol{Q}}^{( - 1)T}$.

Step 5. Simulate **Σ**_*t*_ according to the IW distribution given in Supplementary material (A.4).

Step 6. Simulate **Σ**_*E*_ according to the IW distribution given in Supplementary material (A.5).

Step 7. Simulate ***R***_*e*_ according to the IW distribution given in Supplementary material (A.6).

Step 8. Return to step 1 or terminate when chain length is adequate to meet convergence diagnostics.

The Gibbs sampler for the BMTME Thompson version is similar to the original modified Gibbs sampler except that steps 2 and 3 were replaced for univariate sampling for the transformed random effects (***b***_1_ and ***b***_2_), which are back transformed in Step 4. The advantage of the BMTME Thompson version is that it allows sampling the random effects of ***b***_1_ and ***b***_2_ independently for each trait, which allows improving the speed of the Gibbs sampler because it can be parallelized. However, the remaining parameters (***β***, **Σ**_*t*_, **Σ**_*E*_, and ***R***_*e*_) are sampled exactly as the original Gibbs sampler.

### BMTME_Approx model with SVD

An alternative but only approximate method is to transform the matrix of response variables with SVD as ***Y*** = ***UDV***^*T*^, where ***U*** and ***V*** are orthogonal matrices called the left and right singular vectors, respectively, of dimensions *n* × *n* and *L* × *L*, while ***D*** is a rectangular diagonal matrix of the singular values of order *n* × *L* (where only the first *L* values of ***D*** are positive while the rest are zeros). Therefore, the reparametrized model given in Eq. (2) can be rewritten as:

8 $$\begin{array}{l}\boldsymbol{UDV}^T = {\boldsymbol{X\beta }} + {\boldsymbol{Z}}_1{\boldsymbol{b}}_1 + {\boldsymbol{Z}}_2{\boldsymbol{b}}_2 + {\boldsymbol{e}}\cr \boldsymbol{Y}^ \ast = {\boldsymbol{X\beta }}^ \ast + {\boldsymbol{Z}}_1{\boldsymbol{b}}_1^ \ast + {\boldsymbol{Z}}_2{\boldsymbol{b}}_2^ \ast + {\boldsymbol{e}}^ \ast \end{array}$$

where Y^*^ = ***UD*** = ***YV***, β^*^ = ***βV***, ${\boldsymbol{b}}_1^ \ast$=***b***_1_***V***, ${\boldsymbol{b}}_2^ \ast = {\boldsymbol{b}}_2{\it{V}}$, e^*^ = ***eV***. Y^*^ is of order *n* × *L*, β^*^ is of order *I* × *L*, ${\boldsymbol{b}}_1^ \ast$ is of order *J* × *L*, ${\boldsymbol{b}}_2^ \ast$ is of order *IJ* × *L*, and e^*^ is of order *n* × *L*. Note that the parametrized model does not have the same conceptual definition as the original model (2), first, because β^*^ is a random matrix and not an unknown constant matrix as the matrix of fixed effects, ***β***, and second, because ${\boldsymbol{b}}_1^ \ast$, ${\boldsymbol{b}}_2^ \ast$, and e^*^ are no longer independent. However, if we fix ***V*** as part of the subjacent data structure, and we suppose that **Σ**_*t*_ = ***VD***_*t*1_***V***^*T*^ and **R**_***e***_ = ***VD***_*te*_***V***^*T*^ (they have a restricted parameter space, Lin and Smith (1990)**)**, ${\boldsymbol{b}}_1^ \ast$ is distributed as a matrix variate normal distribution as NM_*J*×*L*_ (**0**, ***G***_*g*_, **D**_*t*1_), ${\boldsymbol{b}}_2^ \ast$ ~ $NM_{JI \times L}\left( {{\mathbf{0}},{\mathbf{\Sigma }}_E \otimes {\boldsymbol{G}}_{\boldsymbol{g}},{\bf{D}}_{t1}} \right)$, and e^*^ ~ NM_*n*×*L*_(**0**, ***I***_*n*_, **D**_*te*_), where ***D***_*t*1_ and ***D***_*te*_ are diagonal variance–covariance matrices of dimension *L* × *L*. It is important to point out that, under this approximate model, the (co)variance matrix for environment is assumed an identity matrix, **Σ**_*E*_ = **I**_*I*_.

To use the existing software, we replaced the distribution of transformed random effects ${\boldsymbol{b}}_2^ \ast$ with ${\boldsymbol{b}}_2^ \ast$ ~ NM_*JI*×*L*_(**0**, **I**_*I*_ ⊗ ***G***_***g***_, **D**_*t*2_), where ***D***_*t*2_ is another diagonal variance–covariance matrix of traits of dimension *L* × *L*. With this, the resulting model (8) can be estimated with the R package BGLR, which is appropriate for univariate analysis. From Eq. (8), it is clear that the parameter estimates and predicted values of the original model (Eq. (2)) without transformation can be approximated as:

9 $$\widehat {\boldsymbol{\beta }} = \widehat {\boldsymbol{\beta }}^ \ast {\it{\boldsymbol{V}}}^T$$

10 $$\widehat {\boldsymbol{b}}_1 = \widehat {\boldsymbol{b}}_1^ \ast {\it{\boldsymbol{V}}}^T$$

11 $$\widehat {\boldsymbol{b}}_2 = \widehat {\boldsymbol{b}}_2^ \ast {\it{\boldsymbol{V}}}^T$$

12 $$\widehat {\boldsymbol{Y}}^ \ast = \widehat {\boldsymbol{Y}}^ \ast {\boldsymbol{{V}}}^T\quad ;\quad {\mathrm{with}}\quad \widehat {\boldsymbol{Y}}^ \ast = {\boldsymbol{X}}\widehat {\boldsymbol{\beta }}^ \ast + {\boldsymbol{Z}}_1\widehat {\boldsymbol{b}}_1^ \ast + {\boldsymbol{Z}}_2\widehat {\boldsymbol{b}}_2^ \ast$$

13 $$\widehat {\bf{\Sigma }}_{t1} = {\mathrm{\bf{V}}}\widehat {\bf{D}}_{t1}{\bf{V}}^T$$

14 $$\widehat {\bf{\Sigma }}_{t2} = {\mathrm{\bf{V}}}\widehat {\bf{D}}_{t2}{\bf{V}}^T$$

15 $$\widehat {\bf{\Sigma }}_{te} = {\mathrm{\bf{V}}}\widehat {\bf{D}}_{te}{\bf{V}}^T$$

### Steps for implementing the proposed BMTME_Approx model

Step 1: De-correlate the original traits with the SVD as ***Y*** = ***UDV***^*T*^ and use it as response variable Y^*^ = ***UD*** = ***YV***.

Step 2: Implement model (1) but using one column at a time of the uncorrelated matrix Y^*^ as the response variable. This means that a total of *L* single analyses are done with the model in Eq. (1).

Step 3: With the output of Step 2, the predicted values can be calculated in terms of the transformed values with $\widehat {\boldsymbol{Y}}^ \ast = {\boldsymbol{X}}\widehat {\boldsymbol{\beta }}^ \ast + {\boldsymbol{Z}}_1\widehat {\boldsymbol{b}}_1^ \ast + {\boldsymbol{Z}}_2\widehat {\boldsymbol{b}}_2^ \ast$. With this new output, we construct the diagonal matrices $\widehat {\bf{D}}_{t1}$, $\widehat {\bf{D}}_{t2}$, and $\widehat {\bf{D}}_{te}$.

Step 4: Finally, with Eq. (12), we obtain the predicted values in terms of the original traits. Furthermore, with Eqs. (9–11, 13–15), we obtain the parameter estimates of the beta coefficients, ***β***, random effects ***b***_1_ and ***b***_2_, as well as the variance–covariance matrices of traits corresponding to traits in the first interaction term, **Σ**_*t*1_, for traits in the second interaction term, **Σ**_*t*2_, and the residual variance–covariance matrix of traits, **R**_*e*_. The R code for implementing this proposed BMTME_Approx model in BGLR is given in Supplementary material.

For implementing the proposed BMTME_Approx model with random cross-validation, we will follow the four-step procedure exactly as described above, except for the first step, which is modified. Now, in Step 1, we will de-correlate the traits in the training data set (***Y***_***t***_) with the SVD as ${\boldsymbol{Y}}_t = {\boldsymbol{U}}_t{\boldsymbol{D}}_t{\it{V}}_t^{\boldsymbol{T}}$, where the subscript *t* denotes that these matrices were estimated with the training data set. Then we transform the response variable into Y^*^ = ***U***_*t*_***D***_*t*_ = ***Y***_*t*_***U***_*t*_, and expand Y^*^ with the number of rows that are the same size as the testing data set; the expanded rows should all be replaced with NA, to represent the missing values. The positions of the expanded rows with missing values will correspond to the testing data set. Once this is done, the remaining steps must be followed exactly as in the above procedure that is described for the full data set. Note that the dimension of the response variable matrix with the training data set has fewer rows than the full data set of response variables.

It is important to point out that the BMTME model was built to estimate only one variance–covariance matrix of traits, **Σ**_*t*_, involved in the two interaction terms, genotype × trait and genotype × environment × trait. However, the BMTME_Approx model allows estimating a variance–covariance matrix of traits for each interaction term in which the traits are involved. The BMTME model is also able to estimate an unstructured variance–covariance matrix for the environments, **Σ**_*E*_; this is not reported for the BMTME_Approx model because an identity matrix is assumed.

### Hyperparameters

First we provide the hyperparameters for the BMTME model: ***β*** ~ *MN*_*I*×*L*_ (***β***_0_, ***I***_*I*_, ***S***_*βt*_), ***b***_1_|**Σ**_*t*_ ~ *MN*_*J*×*L*_ (**0**, ***G***_*g*_, **Σ**_*t*_), ***b***_2_|**Σ**_*t*_, **Σ**_*E*_ ~ *MN*_*IJ*×*L*_ (**0**, **Σ**_*E*_ ⊗ ***G***_*g*_, **Σ**_*t*_), **Σ**_*E*_ ~ IW(*ν*_*E*_ = 5, ***S***_*E*_ = ***S***_*E*_), **Σ**_*t*_ ~ IW(*ν*_*t*_ = 5, ***S***_*t*_ = ***S***_*t*_), and **R**_*e*_ ~ IW(*ν*_*e*_ = 5, ***S***_*e*_ = ***S***_*e*_); ***β***_0_ was obtained as the least square of each trait. The remaining hyperparameters ***S***_*βt*_, ***S***_*t*_, ***S***_*E*_, and ***S***_*e*_ are given in Supplementary material. The hyperparameters for the BMTME_Approx were exactly the same to those of the BMTME, but for the univariate analysis, it were as those used in the BGLR software (Pérez-Rodríguez and de los Campos 2014). The proposed Gibbs sampler was implemented in the R-software (R Core Team 2018). A total of 60,000 iterations were performed with a burn-in of 20,000, so that 40,000 samples were used for inference. To eliminate potential problems due to the autocorrelation function (ACF), we considered a thinning of 5. The convergence of the MCMC chains was monitored using trace plots, ACF and Gelman-Rubin diagnostics. We provide weakly informative priors to implement the proposed models. It is important to point out that the proposed BMTME_Approx model only works for normally distributed traits. However, when there is considerable departure from normality, we suggest using independent component analysis (ICA) instead of SVD for transforming the matrix of response variables (***Y***) (see Supplementary material for its implementation).

### Simulated data set 1 and data set 2

To test the proposed models and methods, we simulated multiple-trait and multiple-environment data using the model in Eq. (2). For this first data set, we used the following parameters: 3 environments, 3 traits, 200 genotypes, and 1 replication of the environment–trait–genotype combination. We assumed that ***β***^*T*^ = [13, 10, 5, 12, 8, 7, 11, 9, 6], where the first three beta coefficients belong to traits 1, 2, and 3 in environment 1, the second three values belong to the three traits in environment 2, and the last three belong to environment 3. We assumed that the GRM is known and equal to ***G***_*g*_ = 0.3***I***_200_ + 0.7***J***_200_, where ***I***_200_ is an identity matrix of order 200 and ***J***_200_ is a matrix of order 200 × 200 of ones. The parameters used for building the GRM were chosen to provide a high-level relationship between lines (Montesinos-López et al. 2016).

Therefore, the total number of observations is 3 × 200 × 3 × 1 = 1800, that is, 600 for each trait. Since a covariance matrix can be expressed in terms of a correlation matrix (***R***_*r*_) and a standard deviation matrix $\left( {{\boldsymbol{D}}_r^{1/2}} \right)$ as: ${\mathbf{\Sigma }}_r = {\boldsymbol{D}}_r^{1/2}{\boldsymbol{R}}_{\boldsymbol{r}}{\boldsymbol{D}}_r^{1/2}$, with *r* = *t*, *E*, *e*, where *r* = *t* represents the genetic covariance between traits, *r* = *E* represents the genetic covariance matrix between environments, and *r* = *e* represents the residual covariance matrix between traits. For the three covariance matrices (*r* = *t*, *E*, *e*), we used ***R***_*r*_ = 0.75***I***_3_ + 0.25***J***_3_, where ***J***_3_ is a matrix of order 3 × 3 of ones, and ${\boldsymbol{D}}_t^{1/2}$ = diag(0.9, 0.8, 0.9), ${\boldsymbol{D}}_E^{1/2}$ = diag(0.5, 0.65, 0.75) and ${\boldsymbol{D}}_e^{1/2}$ = diag(0.6, 0.42, 0.33). For the second data set, the parameters used in the simulation were: ***β***^*T*^ = [13, 12.5, 12, 11.5, 11, 10.5, 10, 12, 11.5, 11, 10.5, 10.5, 10,10,11,11.5,12, 12, 11, 10,10.5], where the first seven beta coefficients belong to traits 1–7 in environment 1, the second seven values to the 7 traits in environment 2, and the last seven belong to environment 3.

The matrix of the relationship between lines was generated as ***G***_*g*_ = 0.3***I***_200_ + 0.7***J***_200_ and was equal to the first simulation data set. Here the total number of observations is 3 × 200 × 7 × 1 = 4200, that is, 600 for each trait. For two of the three covariance matrices (*f* = *t*, *e*), we used

$${\boldsymbol{R}}_f = \left[\begin{array}{*{20}{l}}1.000 & 0.970 & 0.944 & 0.917 & 0.890 & 0.862& 0.833\cr - & 1.000 & 0.925 & 0.899 & 0.872 & 0.844 & 0.816\cr - & - & 1.000 & 0.875 & 0.849 & 0.822 & 0.794\cr - & - & - & 1.000 & 0.825 & 0.798 & 0.772\cr - & - & - & - & 1.000 & 0.775 & 0.748\cr - & - & - & - & - & 1.000 & 0.725\cr - & - & - & - & - & - & 1.000\cr\end{array} \right]\,{\mathrm{and}}$$

${\boldsymbol{D}}_f^{1/2}$ = diag(1, 1.0003, 1.0003, 1, 0.9996, 1, 1.0003), while ${\boldsymbol{R}}_E = {\boldsymbol{D}}_E^{1/2}$ = diag(1, 1, 1), that is, we assumed independence between the environments. It is important to point out that this second data set has a high genetic and environmental correlation between traits.

### Experimental data sets

#### Maize data set

The first real data set used for implementing the proposed model is composed of 309 double-haploid maize lines. Traits available in this data set include grain yield (GY), anthesis-silking interval (ASI), and plant height (PH); each of these traits was evaluated in three optimum rain-fed environments (EBU, KAT, and KTI). After editing, information from 158,281 markers was used. This data set was also used by Montesinos-López et al. (2016) and includes best linear unbiased estimates (BLUEs) obtained based on a mixed model analysis of individual trials of a first analysis.

#### Wheat data set

Here we present information on the second real data set used for implementing the proposed model. This real data set is composed of 250 wheat lines that were extracted from a large set of 39 yield trials grown during the 2013–2014 crop season in Ciudad Obregon, Sonora, Mexico (Rutkoski et al. 2016). The traits under study were days to heading (DH), GY, PH, and the green normalized difference vegetation index (NDVI). Each of these traits was evaluated in three environments (Bed2IR, Bed5IR, and Drip). The marker information used after editing was from 12,083 markers, this data set also used by Montesinos-López et al. (2016); those interested in obtaining more details about this data set can consult this publication. The phenotypes of each trait are BLUEs obtained after a first analysis where they were adjusted by the experimental field design.

#### High-throughput (HTP) data set

This data set belongs to an experiment that used HTP wheat plant phenotyping conducted at Ciudad Obregon, Sonora, México. The data set is comprised of 976 wheat lines that were extracted from a large set of 1170 lines from the CIMMYT Global Wheat Program. The following traits were under study: GY, DH, red normalized difference vegetation index (RNDVI), green normalized difference vegetation index (GNDVI), simple ratio (SRa), ratio analysis of reflectance spectra chlorophyll a (RARSa), ratio analysis of reflectance spectra chlorophyll b (RARSb), ratio analysis of reflectance spectra chlorophyll c (RARSc), normalized pheophytinization index (NPQI), and photochemical reflectance index (PR). Each of these traits was evaluated in three environments (drought, irrigated, and reduced irrigation). After marker editing, information from 1448 markers was used. This data set was also used by Montesinos-López et al. (2017b, c). The phenotypes of each trait are BLUEs obtained after a first analysis where they were adjusted by the experimental field design.

#### Large EYT set

This data set belongs to CIMMYT’s three elite yield trial (EYT) nurseries, consisting of 2505 lines of wheat, genotyped by genotyping-by-sequencing (GBS). These were evaluated for GY, DH, and PH in five environments (BED_5IR, FLAT_5IR, BED_2IR, FLAT_DRIP, and LHT) evaluated in Ciudad Obregon, Mexico, under bed and flat planting systems. The EYT nurseries were sown in 39 trials, each containing 28 lines and two checks that were arranged in an alpha lattice design with three replications and six blocks. The nurseries were evaluated for the three traits under study on a plot basis during 2014 (EYT 13–14), 2015 (EYT 14–15), and 2016 (EYT 15–16). We used BLUEs as observed values of the breeding lines resulting from adjusting for the corresponding experimental design. All the 2505 lines were genotyped using GBS (Elshire et al. 2011; Poland et al. 2012) at Kansas State University, with an Illumina HiSeq2500 for obtaining genome-wide markers. Markers with missing data >60% (minor allele frequency <5% and percentage of heterozygosity >10%) were removed, and we obtained 2038 markers, which were used for the analysis. Also, the traits used are BLUEs obtained after a first analysis where they were adjusted by the experimental field design in each trial.

### Random cross-validation scheme

For testing the prediction ability of the proposed models, the BMTME_Approx model and the BMTME model, we implemented a type of cross-validation where all the traits are missing in some individuals and the information of some individuals is missing in some environments (that is, their lines and traits are missing), but in at least one environment there is information available on those individuals. We implemented a 20 random cross-validation scheme for all the data sets under study, with the exception of the HTP and the large EYT data sets, in which we implemented 10 random cross-validations. For the 20 random cross-validation scheme, in each partition we assigned 20% of the data to the testing set and the remaining 80% of the data to the training set, while for the 10 random cross-validation scheme, we assigned 30% of the data to the testing set and 70% to the training set. The models were fitted with the information in the training data sets, and the prediction accuracy was evaluated with the testing data sets.

The metrics used for reporting the prediction accuracy were the Pearson’s correlation (Cor) and the mean square error of prediction (MSEP) obtained by averaging the information of the 20 (or 10) random partitions resulting from the testing data sets. The models were implemented in the R package (R Core Team 2018) and the proposed BMTME_Approx model can be implemented in the BGLR package of de los Campos and Pérez-Rodríguez (2014). On the other hand, the BMTME model was implemented in R with the model proposed by Montesinos-López et al. (2016).

### Data repository

The phenotypic and genotypic information of the experimental wheat and maize data sets included in this study can be downloaded from the link http://hdl.handle.net/11529/10646 (Montesinos-López et al. 2016). This link includes phenotypic data on maize (Data.maize) and wheat (Data.trigo), as well as genomic data on maize (G.maize) and wheat (G.trigo).

The HTP data and materials used in this study can be downloaded from the link given in Montesinos-Lopez et al. (2017c): http://hdl.handle.net/11529/10693 that contains a file corresponding to the phenotypic and band data for each environment, Drought.Phe and Bands.RData, EarlyHeat.Phe, and Bands.RData, Irrigated.Phe and Bands, RData, Irrigated.Phe and Bands.RData. The large EYT data (Data.EYT.2018) can be downloaded from the link http://hdl.handle.net/11529/10547920.

## Results

The results are described in two main sections: The first section presents the results of the simulated data sets, while the second presents the results of the real data sets. It is important to point out that we do not present results for the BMTME Thompson version because it is only a different reparametrization of the original BMTME model.

### Simulated data sets

#### Data set 1

First, we present the parameter estimates of the BMTME and the BMTME_Approx models. Table 1 shows that the beta coefficients of both models are similar. In general, the beta coefficients of the BMTME model are larger than those of the BMTME_Approx model, the smallest difference is 3% observed in environment 1 and trait 1, and the largest difference is 15.7% observed in environment 3 and trait 3. The variance–covariance matrix of traits (**Σ**_*t*_) of the BMTME model is quite similar to the variance–covariance matrices of the BMTME_Approx model (**Σ**_*t*1_, **Σ**_*t*2_). The variance–covariance components of the residual of both models are quite similar, with the smallest difference (1.6%) observed in trait 2 and trait 1 and the largest difference (33.7%) observed in trait 3 and trait 2. It is worth pointing out again that the BMTME and BMTME_Approx models are different; consequently, we cannot expect the exact same parameter estimates. Finally, when comparing the observed versus the predicted values for each trait using Pearson’s correlation and MSEP, we observed that the BMTME_Approx model produced predicted values that are very similar to those of the BMTME model (see Table 1).

**Table 1:** Parameter estimates (posterior means) of the BMTME model and the BMTME_Approx model for simulated data set 1

	True values			BMTME			BMTME_Approx		
	***β***			$\widehat {\boldsymbol{\beta }}$			$\widehat {\boldsymbol{\beta }}$		
	Trait1	Trait2	Trait3	Trait1	Trait2	Trait3	Trait1	Trait2	Trait3
Env1	13	12	11	14.173	11.516	11.331	13.747	10.982	10.387
Env2	10	8	9	9.514	7.993	9.767	9.173	7.438	8.915
Env3	5	7	6	4.180	6.286	6.540	3.906	5.915	5.512

	**Σ**_*t*_			$\widehat {\bf{\Sigma }}_t$			$\widehat {\bf{\Sigma }}_{t1}$		
	Trait1	Trait2	Trait3	Trait1	Trait2	Trait3	Trait1	Trait2	Trait3
Trait1	0.900	0.212	0.225	0.979	0.297	0.382	0.911	0.266	0.331
Trait2	0.212	0.800	0.212	0.297	1.096	0.288	0.266	0.970	0.128
Trait3	0.225	0.212	0.900	0.382	0.288	0.803	0.331	0.128	0.934

	**Σ**_*E*_			$\widehat {\bf{\Sigma }}_E$			$\widehat {\bf{\Sigma }}_{t2}$		
	Env1	Env2	Env3	Env1	Env2	Env3	Trait1	Trait2	Trait3
Env1	0.500	0.143	0.153	0.374	0.184	0.052	0.571	0.197	0.193
Env2	0.143	0.650	0.175	0.184	0.521	0.201	0.197	0.438	0.216
Env3	0.153	0.175	0.750	0.052	0.201	0.412	0.193	0.216	0.487

	***R***_*e*_			$\widehat {\boldsymbol{R}}_e$			$\widehat {\boldsymbol{R}}_e$		
	Trait1	Trait2	Trait3	Trait1	Trait2	Trait3	Trait1	Trait2	Trait3
Trait1	0.600	0.125	0.111	0.775	0.180	0.202	0.603	0.177	0.171
Trait2	0.125	0.420	0.093	0.180	0.436	0.150	0.177	0.475	0.201
Trait3	0.111	0.093	0.330	0.202	0.150	0.474	0.171	0.201	0.522

				Prediction accuracy			Prediction accuracy		
				Trait1	Trait2	Trait3	Trait1	Trait2	Trait3
Cor	—	—	—	0.986	0.978	0.966	0.989	0.978	0.972
MSEP	—	—	—	0.479	0.247	0.307	0.479	0.247	0.307

Cor and MSEP denote Pearson’s correlation and mean square error of prediction, respectively, between the observed and predicted values. True values denotes the true parameter values used for simulating the data

***β*** denotes the beta coefficients**, Σ**_*t*_ denotes the genetic (co)variance matrix of traits, **Σ**_*E*_ denotes the genetic (co)variance matrix of environments, ***R***_*e*_ denotes the residual (co)variance matrix of traits, and the symbol hat (^) denotes estimates of the corresponding parameters

With regard to prediction accuracy, Table 2 shows that the BMTME model was the best: In six out of the nine trait–environment combinations, it was superior to the BMTME_Approx model in terms of Pearson’s correlation and MSEP. On average, the BMTME model was superior to the BMTME_Approx model by 0.94% and 0.88% in terms of Pearson’s correlation and MSEP, respectively. From the results of these simulated data, it is evident that the BMTME_Approx model is very similar to the BMTME model in terms of prediction accuracy (Table 2).

**Table 2:** Average Pearson’s correlation (Cor) and average mean square error of prediction (MSEP) for each trait–environment combination for simulated data set 1 resulting from the testing set of the 20 random partitions

Trait_Env	BMTE				BMTME_Approx			
	Cor	SE	MSEP	SE	Cor	SE	MSEP	SE
y1_Env1	0.326	0.032	0.714	0.026	**0.338**	0.036	**0.705**	0.026
y2_Env1	**0.515**	0.029	**0.554**	0.021	0.508	0.031	0.561	0.022
y3_Env1	**0.472**	0.026	**0.597**	0.027	0.461	0.025	0.606	0.027
y1_Env2	0.344	0.019	1.062	0.057	**0.351**	0.019	**1.061**	0.057
y2_Env2	**0.546**	0.028	**0.579**	0.027	0.527	0.029	0.608	0.028
y3_Env2	**0.418**	0.032	**0.733**	0.041	0.408	0.033	0.734	0.043
y1_Env3	0.411	0.026	0.978	0.041	**0.418**	0.023	**0.976**	0.040
y2_Env3	**0.529**	0.023	**0.700**	0.029	0.523	0.023	0.714	0.028
y3_Env3	**0.456**	0.019	**0.556**	0.025	0.444	0.019	0.564	0.026
Average	**0.446**	0.026	**0.719**	0.033	0.442	0.026	0.726	0.033

The best predictions for each trait–environment combination are in bold

#### Data set 2

First, we present the parameter estimates of the BMTME and BMTME_Approx models. Table 3 shows that the beta coefficients of both models are similar, and in general, the beta coefficients of the BMTME model are larger than those of the BMTME_Approx model. The smallest difference is 5.15%, which is observed in environment 3 and trait 6, while the largest difference is 12.37%, observed in environment 1 and trait 5. The variance–covariance matrix of traits (**Σ**_*t*_) in the BMTME model is very similar to the variance–covariance matrices of the BMTME_Approx model (**Σ**_*t*1_, **Σ**_*t*2_). The variance–covariance components of the residual of both models are quite similar, with the smallest difference (0.48%) observed in trait 7 and trait 1 and the largest difference (27.38%) observed in the variance of trait 5. It is worth reiterating that, with the BMTME and BMTME_Approx, we should not expect exactly the same parameter estimates, as the models are different. Finally, when comparing the observed versus the predicted values for each trait using Pearson’s correlation and MSEP, the BMTME_Approx model produced predicted values that were slightly better than those of the BMTME model (see Table 3). In terms of Pearson’s correlation, the smallest difference in favor of the BMTME_Approx model was 6.73% observed in trait 6, while the largest difference, also in favor of the BMTME_Approx model, was 14.16% observed in trait 3. In terms of MSEP, the smallest difference was 13.81% in trait 1 and the largest difference was 19.34% in trait 5, both in favor of the BMTME_Approx model.

**Table 3:** Parameter estimates (posterior means) of the BMTME model and the BMTME_Approx model for simulated data set 2

	True values							BMTME							BMTME_Approx						
	***β***							$\widehat {\boldsymbol{\beta }}$							$\widehat {\boldsymbol{\beta }}$						
	Trait1	Trait2	Trait3	Trait4	Trait5	Trait6	Trait7	Trait1	Trait2	Trait3	Trait4	Trait5	Trait6	Trait7	Trait1	Trait2	Trait3	Trait4	Trait5	Trait6	Trait7
Env1	13	12.5	12	11.5	11	10.5	10	13.245	11.618	10.645	11.121	9.486	11.027	11.333	11.899	10.328	9.483	9.979	8.312	9.884	10.400
Env2	12	11.5	11	10.5	10.5	10	10	11.335	9.981	10.750	9.283	8.544	10.888	8.666	10.394	9.056	9.958	8.546	7.551	10.175	8.148
Env3	11	11.5	12	12	11	10	10.5	11.018	9.346	10.925	9.811	10.056	10.963	9.676	10.344	8.714	10.271	9.265	9.167	10.399	9.101

	**Σ**_*t*_							$\widehat {\bf{\Sigma }}_t$							$\widehat {\bf{\Sigma }}_{t1}$						
	Trait1	Trait2	Trait3	Trait4	Trait5	Trait6	Trait7	Trait1	Trait2	Trait3	Trait4	Trait5	Trait6	Trait7	Trait1	Trait2	Trait3	Trait4	Trait5	Trait6	Trait7
Trait1	1.000	0.970	0.944	0.917	0.890	0.862	0.833	0.663	0.620	0.575	0.565	0.577	0.570	0.477	0.796	0.686	0.649	0.633	0.549	0.663	0.630
Trait2	0.970	1.001	0.925	0.899	0.872	0.844	0.816	0.620	0.610	0.547	0.524	0.548	0.546	0.416	0.686	0.636	0.551	0.534	0.474	0.560	0.528
Trait3	0.944	0.925	1.001	0.875	0.848	0.822	0.794	0.575	0.547	0.578	0.495	0.550	0.528	0.391	0.649	0.551	0.718	0.559	0.532	0.635	0.523
Trait4	0.917	0.899	0.875	1.000	0.824	0.799	0.772	0.565	0.524	0.495	0.607	0.488	0.488	0.448	0.633	0.534	0.559	0.635	0.472	0.550	0.528
Trait5	0.890	0.872	0.848	0.824	0.999	0.774	0.748	0.577	0.548	0.550	0.488	0.691	0.539	0.369	0.549	0.474	0.532	0.472	0.580	0.524	0.460
Trait6	0.862	0.844	0.822	0.799	0.774	1.000	0.725	0.570	0.546	0.528	0.488	0.539	0.634	0.338	0.663	0.560	0.635	0.550	0.524	0.755	0.550
Trait7	0.833	0.816	0.794	0.772	0.748	0.725	1.001	0.477	0.416	0.391	0.448	0.369	0.338	0.631	0.630	0.528	0.523	0.528	0.460	0.550	0.665

	**Σ**_*E*_							$\widehat {\bf{\Sigma }}_E$							$\widehat {\bf{\Sigma }}_{t2}$						
	Env1	Env2	Env3					Env1	Env2	Env3	—	—	—	—	Trait1	Trait2	Trait3	Trait4	Trait5	Trait6	Trait7
Env1	1	0	0	—	—	—	—	0.649	0.261	−0.026	—	—	—	—	0.715	0.620	0.582	0.564	0.481	0.592	0.546
Env2	0	1	0	—	—	—	—	0.261	0.681	0.033	—	—	—	—	0.620	0.582	0.497	0.478	0.413	0.497	0.447
Env3	0	0	1	—	—	—	—	−0.026	0.033	0.449	—	—	—	—	0.582	0.497	0.648	0.493	0.465	0.560	0.466
	—	—	—	—	—	—	—	—	—	—	—	—	—	—	0.564	0.478	0.493	0.573	0.430	0.485	0.462
	—	—	—	—	—	—	—	—	—	—	—	—	—	—	0.481	0.413	0.465	0.430	0.555	0.439	0.423
	—	—	—	—	—	—	—	—	—	—	—	—	—	—	0.592	0.497	0.560	0.485	0.439	0.701	0.495
	—	—	—	—	—	—	—	—	—	—	—	—	—	—	0.546	0.447	0.466	0.462	0.423	0.495	0.626

	***R***_*e*_							$\widehat {\boldsymbol{R}}_e$							$\widehat {\boldsymbol{R}}_e$						
	Trait1	Trait2	Trait3	Trait4	Trait5	Trait6	Trait7	Trait1	Trait2	Trait3	Trait4	Trait5	Trait6	Trait7	Trait1	Trait2	Trait3	Trait4	Trait5	Trait6	Trait7
Trait1	1.000	0.970	0.944	0.917	0.890	0.862	0.833	1.364	1.340	1.297	1.211	1.270	1.164	1.162	1.492	1.274	1.262	1.196	1.063	1.280	1.167
Trait2	0.970	1.001	0.925	0.899	0.872	0.844	0.816	1.340	1.389	1.281	1.193	1.255	1.150	1.147	1.274	1.177	1.084	1.018	0.919	1.084	0.974
Trait3	0.944	0.925	1.001	0.875	0.848	0.822	0.794	1.297	1.281	1.368	1.169	1.216	1.112	1.107	1.262	1.084	1.320	1.055	0.975	1.158	1.046
Trait4	0.917	0.899	0.875	1.000	0.824	0.799	0.772	1.211	1.193	1.169	1.267	1.140	1.049	1.027	1.196	1.018	1.055	1.170	0.927	1.071	0.980
Trait5	0.890	0.872	0.848	0.824	0.999	0.774	0.748	1.270	1.255	1.216	1.140	1.450	1.082	1.108	1.063	0.919	0.975	0.927	1.053	0.961	0.906
Trait6	0.862	0.844	0.822	0.799	0.774	1.000	0.725	1.164	1.150	1.112	1.049	1.082	1.340	0.981	1.280	1.084	1.158	1.071	0.961	1.394	1.086
Trait7	0.833	0.816	0.794	0.772	0.748	0.725	1.001	1.162	1.147	1.107	1.027	1.108	0.981	1.323	1.167	0.974	1.046	0.980	0.906	1.086	1.208

									Prediction accuracy						Prediction accuracy						
								Trait1	Trait2	Trait3	Trait4	Trait5	Trait6	Trait7	Trait1	Trait2	Trait3	Trait4	Trait5	Trait6	Trait7
—	—	—	—	—	—	—	Cor	0.717	0.697	0.672	0.704	0.709	0.727	0.756	0.773	0.770	0.783	0.771	0.784	0.835	0.811
—	—	—	—	—	—	—	MSEP	1.145	1.183	1.170	1.062	1.221	1.131	1.112	1.006	1.009	0.987	0.923	1.023	0.969	0.933

Cor and MSEP denote Pearson’s correlation and mean square error of prediction, respectively, between the observed and predicted values. True values denotes the true parameter values used for simulating the data. ***β*** denotes the beta coefficients, **Σ**_*t*_ denotes the genetic (co)variance matrix of traits, **Σ**_*E*_ denotes the genetic (co)variance matrix of environments, ***R***_*e*_ denotes the residual (co)variance matrix of traits, and the symbol hat (^) denotes estimates of the corresponding parameters

Table 4 shows that the proposed approximate model, BMTME_Approx, was better than the BMTME model in terms of Pearson’s correlation, as shown in 14 out of the 21 trait–environment combinations. However, in terms of MSEP, the BMTME model was superior to the the BMTME_Approx model, as exemplified by 12 out of the 21 trait–environment combinations. On average, in terms of Pearson’s correlation, the BMTME_Approx model was better than the BMTME model by 7.24%, while in terms of MSEP, both models were, on average, almost identical.

**Table 4:** Average Pearson’s correlation (Cor) and average mean square error of prediction (MSEP) for each trait–environment combination for simulated data set 2 resulting from the testing set of the 20 random partitions

Trait_Env	BMTME				BMTME_Approx			
	Cor	SE	MSEP	SE	Cor	Se	MSEP	SE
y1_1	0.279	0.035	**1.461**	0.049	**0.323**	0.036	1.469	0.047
y2_1	0.251	0.035	**1.473**	0.047	**0.287**	0.036	1.477	0.043
y3_1	0.278	0.032	1.486	0.066	**0.324**	0.029	**1.472**	0.060
y4_1	0.251	0.032	**1.408**	0.051	**0.288**	0.031	1.410	0.050
y5_1	0.272	0.038	**1.721**	0.074	**0.298**	0.040	1.720	0.072
y6_1	0.269	0.038	**1.311**	0.065	**0.275**	0.037	1.313	0.063
y7_1	0.227	0.034	1.419	0.061	**0.261**	0.033	**1.411**	0.059
y1_2	0.221	0.024	1.666	0.095	**0.265**	0.024	**1.648**	0.091
y2_2	0.203	0.026	1.608	0.089	**0.258**	0.024	**1.584**	0.085
y3_2	0.156	0.027	1.637	0.095	**0.158**	0.028	**1.628**	0.093
y4_2	0.237	0.024	1.515	0.069	**0.255**	0.023	**1.513**	0.066
y5_2	**0.226**	0.031	1.681	0.084	0.221	0.034	**1.680**	0.079
y6_2	0.166	0.032	1.904	0.069	**0.188**	0.034	**1.882**	0.065
y7_2	**0.314**	0.029	**1.572**	0.100	0.306	0.025	1.606	0.101
y1_3	**0.076**	0.036	**1.753**	0.077	0.068	0.034	1.763	0.077
y2_3	**0.057**	0.035	**1.818**	0.074	0.051	0.033	1.824	0.075
y3_3	**0.080**	0.031	**1.661**	0.077	0.067	0.031	1.677	0.077
y4_3	**0.107**	0.043	**1.636**	0.066	0.100	0.042	1.648	0.064
y5_3	0.103	0.032	**1.780**	0.068	**0.109**	0.035	1.784	0.067
y6_3	0.065	0.041	**1.761**	0.091	**0.070**	0.036	1.762	0.090
y7_3	**0.119**	0.034	1.680	0.050	0.096	0.032	**1.700**	0.052
Average	0.189	0.033	**1.617**	0.072	**0.203**	0.032	1.618	0.070

The best predictions for each trait–environment combination are in bold

### Experimental data sets

#### Maize data set

First, we compared the parameter estimates of the two models (BMTME and BMTME_Approx). Table 5 shows that the beta coefficients of the proposed BMTME_Approx model are all similar to those of the BMTME model. The variance–covariance matrix of traits (**Σ**_*t*_) in the BMTME model is quite similar to the variance–covariance matrices of the BMTME_Approx model (**Σ**_*t*1_,**Σ**_*t*2_). When comparing the variance–covariance components of the residual of both models, we observe that 6 out of the 9 terms are not significantly different; however, the remaining 3 terms are quite different, as they belong to the covariance of trait PH with the other traits. Finally, when comparing the observed versus the predicted values for each trait using Pearson’s correlation and MSEP, we see that the BMTME_Approx model is slightly better, since in Pearson’s correlation, it outperformed the BMTME model in 2 out of the 3 traits and, on average, the BMTME_Approx model was 2.1% (for trait GY) and 1.4% (for trait ASI) better than the BMTME model. In terms of MSEP, the BMTME_Approx model was better than the BMTME model in 2 out of the 3 traits, and, on average, the BMTME_Approx model was 7% better (for trait GY) and 4.11% better (for trait ASI) than the BMTME model (see Table 5). In general, the parameter estimates and predictions are relatively similar in this data set.

**Table 5:** Parameter estimates (posterior means) of the BMTME model and the BMTME_Approx model for the experimental maize data set

	BMTME			BMTME_Approx		
	$\widehat {\boldsymbol{\beta }}$			$\widehat {\boldsymbol{\beta }}$		
Evu	GY	ASI	PH	GY	ASI	PH
EBU	6.554	2.051	2.444	6.419	1.903	2.342
KAK	5.107	1.288	2.136	4.942	1.203	2.053
KTI	6.213	2.487	2.415	6.068	2.337	2.317

	$\widehat {\bf{\Sigma }}_t$			$\widehat {\bf{\Sigma }}_{t1}$		
Trait^a^	GY	ASI	PH	GY	ASI	PH
GY	0.801	−0.167	0.044	1.205	−0.203	0.365
ASI	−0.167	0.843	−0.031	−0.203	1.890	0.078
PH	0.044	−0.031	0.022	0.365	0.078	0.248

	$\widehat {\bf{\Sigma }}_E$			$\widehat {\bf{\Sigma }}_{t2}$		
Env	EBU	KAK	KTI	GY	ASI	PH
EBU	1.424	0.518	0.604	0.814	−0.041	0.236
KAK	0.518	2.079	1.812	−0.041	0.962	0.056
KTI	0.604	1.812	2.687	0.236	0.056	0.217

	$\widehat {\boldsymbol{R}}_e$			$\widehat {\boldsymbol{R}}_e$		
Trait	GY	ASI	PH	GY	ASI	PH
GY	0.540	−0.080	0.022	0.403	−0.041	0.119
ASI	−0.080	0.526	−0.011	−0.041	0.546	0.027
PH	0.022	−0.011	0.012	0.119	0.027	0.098

	Prediction accuracy			Prediction accuracy		
	GY	ASI	PH	GY	ASI	PH
Cor	0.803	0.790	0.880	0.820	0.802	0.877
MSEP	0.435	0.419	0.009	0.405	0.402	0.010

^a^Traits: grain yield (GY), anthesis silking interval (ASI), and plant height (PH)

Cor and MSEP denote Pearson’s correlation and mean square error of prediction, respectively, between the observed and predicted values. ***β*** denotes the beta coefficients, **Σ**_*t*_ denotes the genetic (co)variance matrix of traits, **Σ**_*E*_ denotes the genetic (co)variance matrix of environments, ***R***_*e*_ denotes the residual (co)variance matrix of traits, and the symbol hat (^) denotes estimates of the corresponding parameters

Next, in Table 6, we see that the proposed approximate model, BMTME_Approx, was better than the BMTME model in terms of Pearson’s correlation, as shown in 5 out of the 9 trait–environment combinations. However, in terms of MSEP, the BMTME model was superior to the BMTME_Approx model in 7 out of the 9 trait–environment combinations. On average, the BMTME_Approx model was better than the BMTME model by 11.72% in terms of Pearson’s correlation, while conversely, the BMTME model was better than the BMTME_Approx by 37.94% (Table 6) in terms of MSEP.

**Table 6:** Average Pearson’s correlation (Cor) and average mean square error of prediction (MSEP) for each trait–environment combination for the experimental maize data set resulting from the testing set of the 20 random partitions

	BMTME				BMTME_Approx			
Env-Trait^a^	Cor	SE	MSEP	SE	Cor	SE	MSEP	SE
EBU_GY	0.327	0.019	0.787	0.019	**0.338**	0.036	**0.705**	0.026
EBU_ASI	0.508	0.016	**0.392**	0.012	0.508	0.031	0.561	0.022
EBU_PH	0.311	0.023	**0.014**	0.003	**0.461**	0.025	0.606	0.027
KAK_GY	**0.406**	0.024	**0.442**	0.021	0.351	0.019	1.061	0.057
KAK_ASI	0.397	0.015	0.937	0.044	**0.527**	0.029	**0.608**	0.028
KAK_PH	**0.476**	0.027	**0.011**	0.001	0.408	0.033	0.734	0.043
KTI_GY	0.292	0.019	**0.841**	0.023	**0.418**	0.023	0.976	0.040
KTI_ASI	0.295	0.017	**0.610**	0.018	**0.523**	0.023	0.714	0.028
KTI_PH	**0.502**	0.018	**0.018**	0.001	0.444	0.019	0.564	0.026
Average	0.390	0.020	**0.450**	0.016	**0.442**	0.026	0.726	0.033

The best predictions for each trait–environment combination are in bold

^a^Traits: grain yield (GY), anthesis silking interval (ASI), and plant height (PH)

#### Wheat data set

First, we compared the parameter estimates of the two models (BMTME and BMTME_Approx) and then their prediction accuracy. Table 7 shows that the beta coefficients of the proposed BMTME_Approx model are similar to those of the BMTME in 9 out of the 12 parameters; however, there are substantial differences in three of the beta coefficients corresponding to trait NDVI. The variance–covariance matrix of traits (**Σ**_*t*_) in the BMTME model is significantly different from the variance–covariance matrices of the BMTME_Approx model (**Σ**_*t*1_, **Σ**_*t*2_).

**Table 7:** Parameter estimates (posterior means) of the BMTME model and the BMTME_Approx model for the experimental wheat data set

	BMTME				BMTME_Approx			
Env	$\widehat {\boldsymbol{\beta }}$				$\widehat {\boldsymbol{\beta }}$			
	DH	NDVI	GY	PH	DH	NDVI	GY	PH
Bed2IR	−3.164	0.060	−0.075	−4.496	−3.202	−0.004	−0.138	−4.647
Bed5IR	−4.026	0.053	−0.284	−7.535	−4.023	−0.011	−0.340	−7.436
Drip	−0.272	0.070	−0.342	−0.511	−0.314	0.006	−0.409	−0.692

	$\widehat {\bf{\Sigma }}_t$				$\widehat {\bf{\Sigma }}_{t1}$			
Trait^a^	DH	NDVI	GY	PH	DH	NDVI	GY	PH
DH	3.440	0.005	−0.098	−1.125	24.552	0.042	−0.566	−11.455
NDVI	0.005	0.000	0.000	−0.002	0.042	0.000	−0.001	−0.019
GY	−0.098	0.000	0.011	0.047	−0.566	−0.001	0.059	0.377
PH	−1.125	−0.002	0.047	1.872	−11.455	−0.019	0.377	8.532

	$\widehat {\bf{\Sigma }}_E$				$\widehat {\bf{\Sigma }}_{t2}$			
Env	Bed2IR	Bed5IR	Drip		DH	NDVI	GY	PH
Bed2IR	7.426	7.265	6.505	—	4.247	0.007	0.059	2.376
Bed5IR	7.265	7.619	6.361	—	0.007	0.000	0.000	0.004
Drip	6.505	6.361	6.388	—	0.059	0.000	0.048	0.256
	—	—	—	—	2.376	0.004	0.256	7.556

	$\widehat {\boldsymbol{R}}_e$				$\widehat {\boldsymbol{R}}_e$			
Trait	DH	NDVI	GY	PH	DH	NDVI	GY	PH
DH	4.762	0.002	0.131	1.778	6.304	0.011	0.042	2.257
NDVI	0.002	0.000	0.000	0.002	0.011	0.000	0.000	0.004
GY	0.131	0.000	0.109	0.531	0.042	0.000	0.083	0.323
PH	1.778	0.002	0.531	18.064	2.257	0.004	0.323	9.443

	Prediction accuracy				Prediction accuracy			
	DH	NDVI	GY	PH	DH	NDVI	GY	PH
Cor	0.9604	0.9953	0.7467	0.7945	0.9699	0.9748	0.9112	0.9308
MSEP	2.7257	0.0000	0.0866	14.1430	2.2085	0.0000	0.0442	6.0107

Cor and MSEP denote Pearson’s correlation and mean square error of prediction, respectively, between the observed and predicted values. ***β*** denotes the beta coefficients, **Σ**_*t*_ denotes the genetic (co)variance matrix of traits, **Σ**_*E*_ denotes the genetic (co)variance matrix of environments, ***R***_*e*_ denotes the residual (co)variance matrix of traits, and the symbol hat (^) denotes estimates of the corresponding parameters

^a^Traits: days to heading (DH), grain yield (GY), plant height (PH), and the green normalized difference vegetation index (NDVI). Each of these traits was evaluated in three environments (Bed2IR, Bed5IR, and Drip)

When comparing the variance–covariance components of the residual of both models, it is evident that 9 out of the 16 terms are not notably different, while the remaining 7 terms are. These very different terms belong to the covariance of trait NDVI with the other traits. Finally, when we compared the models in terms of the observed versus the predicted values for each trait using Pearson’s correlation and MSEP, we saw that the BMTME_Approx model was marginally better, and it was superior to the BMTME model for Pearson’s correlation in 3 out of the 4 traits and, on average, 0.99% better for trait DH, 22.04% better for trait GY, and 17.16% better for trait PH. Furthermore, for MSEP, the BMTME_Approx model was better than the BMTME model in 3 out of the 4 traits and, on average, 18.88% better for trait DH, 48.85% better for trait GY, and 57.5% better for trait PH (see Table 7).

When comparing both models in terms of prediction accuracy with only the testing set of the 20 random partitions implemented, Table 8 shows that the proposed alternative model, BMTME_Approx, was better than the BMTME model in 8 out of the 12 trait–environment combinations in terms of Pearson’s correlation and in 7 out of the 12 trait–environment combinations in terms of MSEP. On average, the BMTME_Approx model was also better than the BMTME model by 2.1% in terms of Pearson’s correlation and by 6.87% in terms of MSEP (Table 8).

**Table 8:** Average Pearson’s correlation (Cor) and average mean square error of prediction (MSEP) for each trait–environment combination for the experimental wheat data set resulting from the testing set of the 20 random partitions

	BMTME				BMTME_Approx			
Trait-Env^a^	Cor	SE	MSEP	SE	Cor	SE	MSEP	SE
DH_Bed2IR	**0.889**	0.009	**7.450**	0.717	0.875	0.011	8.877	0.822
NDVI_Bed2IR	0.843	0.008	0.000	0.000	**0.849**	0.006	0.000	0.000
GY_Bed2IR	0.640	0.013	0.056	0.002	**0.677**	0.013	**0.051**	0.002
PH_Bed2IR	0.641	0.013	23.252	0.804	**0.693**	0.014	**20.911**	0.814
DH_Bed5IR	**0.874**	0.006	**12.446**	0.598	0.853	0.008	13.762	0.664
NDVI_Bed5IR	**0.800**	0.010	0.000	0.000	0.779	0.009	0.000	0.000
GY_Bed5IR	0.178	0.021	0.253	0.008	**0.200**	0.022	**0.238**	0.007
PH_Bed5IR	0.086	0.015	24.200	0.599	**0.170**	0.015	**18.721**	0.539
DH_Drip	**0.925**	0.005	4.575	0.286	0.905	0.005	**4.532**	0.188
NDVI_Drip	0.710	0.012	0.000	0.000	**0.711**	0.014	0.000	0.000
GY_Drip	0.649	0.012	0.127	0.005	**0.678**	0.014	**0.119**	0.004
PH_Drip	0.655	0.018	21.457	0.543	**0.669**	0.019	**20.157**	0.570
Average	0.657	0.012	7.818	0.297	**0.672**	0.012	**7.281**	0.301

^a^Traits: days to heading (DH), grain yield (GY), plant height (PH), and the green normalized difference vegetation index (NDVI). Each of these traits was evaluated in three environments (Bed2IR, Bed5IR, and Drip)

The best predictions for each trait–environment combination are in bold

#### HTP data set

In Table 9 we provide the parameter estimates of the HTP data set, which has 10 traits evaluated in 3 environments, with 976 lines evaluated in each environment. The estimates of the beta coefficients are reasonable since they are consistent with the sample average for each trait in each environment, which is equivalent to the least square estimates.

**Table 9:** Parameter estimates (posterior means of beta coefficients, genetic and residual correlation) of the BMTME_Approx model for the experimental HTP data set

	$$\widehat {\boldsymbol{\beta }}$$									
Env	GY	DH	RNDVI	GNDVI	SRa	RARSa	RARSb	RARSc	NPQI	PR
Drought	2.172	77.408	−0.071	−0.085	−0.092	−0.163	−0.184	−0.200	−0.140	−0.198
Irrigated	6.522	85.728	−0.093	−0.085	−0.083	−0.106	−0.125	−0.132	−0.106	−0.170
Red_Irrig	3.735	81.946	−0.084	−0.088	−0.092	−0.136	−0.163	−0.186	−0.127	−0.195

	**Genetic correlation between traits (upper diagonal corresponds to** $\widehat {\bf{\Sigma }}_{t1}$ **, while lower diagonal to** $\widehat {\bf{\Sigma }}_{t2}$ **)**									
Trait^a^	GY	DH	RNDVI	GNDVI	SRa	RARSa	RARSb	RARSc	NPQI	PR
GY	1.000	**0.781**	−**0.612**	−0.421	−0.340	−0.251	−0.275	−0.265	−0.302	−0.437
DH	0.154	1.000	−**0.708**	−**0.576**	−0.495	−0.467	−0.489	−0.483	−0.499	−**0.658**
RNDVI	−0.271	−0.248	1.000	**0.860**	**0.827**	**0.776**	**0.767**	**0.707**	**0.811**	**0.799**
GNDVI	0.123	−0.260	0.276	1.000	**0.972**	**0.934**	**0.929**	**0.886**	**0.949**	**0.914**
SRa	0.256	−0.263	0.429	0.463	1.000	**0.944**	**0.944**	**0.898**	**0.970**	**0.899**
RARSa	**0.605**	−0.231	−0.170	0.493	0.441	1.000	**0.992**	**0.971**	**0.983**	**0.949**
RARSb	**0.667**	−0.284	−0.040	0.350	**0.547**	**0.817**	1.000	**0.985**	**0.988**	**0.967**
RARSc	**0.648**	−0.262	−0.050	0.260	0.417	**0.681**	**0.846**	1.000	**0.957**	**0.970**
NPQI	**0.512**	−0.274	0.196	0.260	**0.659**	**0.578**	**0.825**	**0.658**	1.000	**0.949**
PR	0.485	−0.402	0.070	0.352	0.411	**0.595**	**0.786**	**0.830**	**0.629**	1.000

	**Residual correlation between traits**									
Trait	GY	DH	RNDVI	GNDVI	SRa	RARSa	RARSb	RARSc	NPQI	PR
GY	1.000	0.164	−0.210	0.086	0.187	**0.508**	**0.548**	**0.539**	0.402	0.382
DH	—	1.000	−0.199	−0.201	−0.207	−0.206	−0.248	−0.231	−0.229	−0.339
RNDVI	—	—	1.000	0.281	0.361	−0.051	0.026	0.023	0.184	0.108
GNDVI	—	—	—	1.000	0.406	0.430	0.315	0.264	0.226	0.348
SRa	—	—	—	—	1.000	0.425	**0.500**	0.391	**0.572**	0.354
RARSa Env	—	—	—	—	—	1.000	**0.765**	**0.642**	**0.548**	**0.546**
RARSb	—	—	—	—	—	—	1.000	**0.777**	**0.765**	**0.707**
RARSc	—	—	—	—	—	—	—	1.000	**0.581**	**0.749**
NPQI	—	—	—	—	—	—	—	—	1.000	**0.541**
PR	—	—	—	—	—	—	—	—	—	1.000

	**Prediction accuracy**									
	GY	DH	RNDVI	GNDVI	SRa	RARSa	RARSb	RARSc	NPQI	PR
Cor	0.970	0.958	0.797	0.612	0.709	0.928	0.958	0.961	0.867	0.872
MSEP	0.206	2.108	0.000	0.000	0.000	0.000	0.000	0.000	0.000	0.000

^a^Traits are: grain yield (GY), days to heading (DH), red normalized difference vegetation index (RNDVI), green normalized difference vegetation index (GNDVI), simple ratio (SRa), ratio analysis of reflectance spectra chlorophyll a (RARSa), ratio analysis of reflectance spectra chlorophyll b (RARSb), ratio analysis of reflectance spectra chlorophyll c (RARSc), normalized pheophytinization index (NPQI), and photochemical reflectance index (PR)

Cor and MSEP denote Pearson’s correlation and mean square error of prediction, respectively, between the observed and predicted values. Environments are drought, irrigated, and reduced irrigation (Red_Irrig). In bold are the correlations >0.5. ***β*** denotes the beta coefficients, **Σ**_*t*_ denotes the genetic (co)variance matrix of traits, **Σ**_*E*_ denotes the genetic (co)variance matrix of environments, ***R***_*e*_ denotes the residual (co)variance matrix of traits, and the symbol hat (^) denotes estimates of the corresponding parameters

Rather than reporting the variance–covariance of traits, we report the correlation between traits, which can be more useful, as it gives a better idea of the level of correlation between traits in the whole data set. Our proposed BMTME_Approx model estimates two variance–covariance matrices for the genetic part of the traits and one for the residual part of the traits; in this vein, we report two correlation matrices for the genetic part of the traits and one for the residual correlation of the traits. In the section “genetic correlation between traits” in Table 9, the upper diagonal part gives the correlations between traits corresponding to the term genotype × trait, where we can observe that 33 (73.33%) of the 45 possible correlations were, in absolute values, >0.5. On the other hand, in the lower diagonal part of the genetic correlation section are given the genetic correlations that correspond to the three-way interaction term environment × genotype × trait. Here we can observe that only 16 (35.56%) of the 45 possible correlations are >0.5.

In general, there is evidence of a reasonably high genetic correlation between traits in this HTP data set. In Table 9, the section on the residual correlation between traits shows that only 15 (33.33%) out of the 45 possible residual correlations are >0.5. This information indicates that the phenotypic correlation between the ten traits is more influenced by the genetic part than by the residual part. Finally, the “prediction accuracy” section of Table 9 shows the Pearson’s correlation and MSEP obtained for the whole data set between the observed and predicted values for each trait. In general, all the observed Pearson’s correlations are >0.86, with the exception of the RNDVI, GNDVI, and SRa traits, which had a Pearson’s correlation <0.8.

The prediction accuracies for the HTP data set are given in Table 10. The predictions reported were the average of the ten random partitions of the testing data set. Here we only report the prediction accuracy for the BMTME_Approx model since it is extremely difficult in terms of implementation time to run the BMTME_model due to the large data set. Table 10 shows that the best predictions in terms of Pearson’s correlation were observed for trait DH, while the predictions for the rest of the traits in general were low. Also, in terms of Pearson’s correlation, the best predictions were observed in the irrigated environment. On the other hand, in terms of MSEP for traits GY and DH, the best predictions were observed in the drought environment; however, there were no significant differences in terms of prediction accuracy between the remaining traits, since in all traits, the MSE was almost zero.

**Table 10:** Average Pearson’s correlation (Cor) and average mean square error of prediction (MSEP) for each trait–environment combination for the experimental HTP data set resulting from the testing set of the 10 random partitions

	Drought		Irrigated		Reduced Irrigated	
Trait^a^	Cor	SE	Cor	SE	Cor	SE
GY	0.080	0.009	−0.054	0.007	**0.152**	0.006
DH	**0.812**	0.005	0.734	0.005	0.771	0.004
RNDVI	0.085	0.009	**0.160**	0.008	0.027	0.005
GNDVI	0.150	0.012	**0.290**	0.012	0.047	0.011
SRa	0.179	0.007	**0.276**	0.011	0.202	0.012
RARSa	0.167	0.007	**0.304**	0.015	0.150	0.010
RARSb	0.224	0.012	**0.326**	0.017	0.238	0.015
RARSc	0.195	0.015	**0.391**	0.013	0.078	0.009
NPQI	0.135	0.010	**0.237**	0.007	0.141	0.011
PR	−0.062	0.010	**0.342**	0.020	0.046	0.007

Trait	Drought		Irrigated		Reduced Irrigated	
	MSEP	SE	MSEP	SE	MSEP	SE
GY	**0.298**	0.005	0.464	0.005	0.143	0.002
DH	**3.695**	0.169	11.726	0.163	4.941	0.072
RNDVI	0.000	0.000	0.000	0.000	0.000	0.000
GNDVI	0.000	0.000	0.000	0.000	0.000	0.000
SRa	0.000	0.000	0.000	0.000	0.000	0.000
RARSa	0.000	0.000	0.000	0.000	0.000	0.000
RARSb	0.000	0.000	0.000	0.000	0.000	0.000
RARSc	0.000	0.000	0.000	0.000	0.000	0.000
NPQI	0.000	0.000	0.000	0.000	0.000	0.000
PR	0.000	0.000	0.000	0.000	0.000	0.000

The best predictions for each trait in the three environments are in bold

^a^Traits are: grain yield (GY), days to heading (DH), red normalized difference vegetation index (RNDVI), green normalized difference vegetation index (GNDVI), simple ratio (SRa), ratio analysis of reflectance spectra chlorophyll a (RARSa), ratio analysis of reflectance spectra chlorophyll b (RARSb), ratio analysis of reflectance spectra chlorophyll c (RARSc), normalized pheophytinization index (NPQI), and photochemical reflectance index (PR)

#### Large EYT data set

In Table 11, we provide the parameter estimates of the EYT data set that has 3 traits evaluated in 5 environments, with 2505 lines evaluated in each environment. We found that the estimates of the beta coefficients are reasonable since they are consistent with the least square estimates.

**Table 11:** Parameter estimates (posterior means of beta coefficients, genetic and residual correlation) of the BMTME_Approx model for the experimental large EYT data set

	$$\widehat {\boldsymbol{\beta }}$$					
Trait	BED_5IR	FLAT_5IR	BED_2IR	FLAT_DRIP	LHT	
GY	6.349	6.407	3.792	2.130	3.302	—
DH	81.810	79.538	80.628	75.410	58.412	—
PH	102.856	102.766	84.986	73.347	68.810	—

	^a^Genetic correlation between traits			Residual correlation between traits		
Trait	GY	DH	Height	GY	DH	Height
GY	1.000	−0.487	0.599	1.000	−0.393	0.580
DH	−0.086	1.000	−0.421	—	1.000	−0.178
PH	0.631	0.403	1.000	—	—	1.000

	Cor			MSEP		
	GY	DH	Height	GY	DH	Height
Prediction accuracy	0.928	0.894	0.907	0.489	19.688	46.073

Cor and MSEP denote Pearson’s correlation and mean square error of prediction, respectively, between the observed and predicted values

^a^Genetic correlation between traits (the upper diagonal corresponds to $\widehat {\bf{\Sigma }}_{t1}$, while the lower diagonal is for $\widehat {\bf{\Sigma }}_{t2}$). Traits are grain yield (GY), days to heading (DH), and plant height (PH)

Here we also report the correlation between traits to have a better idea of the level of correlation between traits in the whole data set. Furthermore, we report two matrices of correlation for the genetic part of traits and one for the residual correlation of traits. In the section “genetic correlation between traits” in Table 11, the upper diagonal part gives the correlations between traits corresponding to the term genotype × trait, where we can observe that the correlation between PH and DH was the only one >0.5. On the other hand, in the lower diagonal part of the genetic correlation section, between traits are given the genetic correlations that correspond to the three-way interaction term environment × genotype × trait. Here we can observe that only the correlation between GY and PH was >0.5 (Table 11).

In Table 11, in the section on the residual correlation between traits, we can observe that only the correlation between GY and PH was >0.5. Finally, in the “prediction accuracy” section of Table 11, we can observe the Pearson’s correlation and MSEP obtained for the whole data set between the observed and predicted values for each trait. In general, all the observed Pearson’s correlations are >0.89.

The prediction accuracies for this large wheat data set are given in Table 12. The predictions reported are the average of the ten random partitions of the testing data set. Here we only report the prediction accuracy for the BMTME_Approx model; owing to the size of the data set, it almost impossible to run the BMTME_model. Table 12 shows that the best predictions in terms of Pearson’s correlation were for trait GY, while the worst were for trait DH. At the same time, the best predictions for GY, DH, and PH in terms of Pearson’s correlation were observed in environments BED_5IR, FLAT_5IR, and BED_2IR, respectively. On the other hand, in terms of MSEP for traits GY, DH, and PH, the best predictions were observed in BED_2IR, LHT, and FLAT_5IR, respectively (Table 12).

**Table 12:** Average Pearson’s correlation (Cor) and average mean square error of prediction (MSEP) for each trait–environment combination for the experimental large EYT data set resulting from the testing set of the 10 random partitions

	GY		DH		PH	
Env	Cor	SE	Cor	SE	Cor	SE
BED_5IR	**0.417**	0.012	0.292	0.014	0.110	0.013
FLAT_5IR	0.274	0.012	**0.330**	0.017	0.276	0.009
BED_2IR	0.247	0.015	0.230	0.015	**0.408**	0.014
FLAT_DRIP	0.259	0.013	0.259	0.012	0.371	0.017
LHT	0.341	0.011	0.278	0.006	0.213	0.009
Average	0.308	0.013	0.278	0.013	0.276	0.012

Env	MSEP	SE	MSEP	SE	MSEP	SE
BED_5IR	0.528	0.008	31.793	0.513	24.620	0.321
FLAT_5IR	0.514	0.008	18.144	0.272	**23.882**	0.435
BED_2IR	**0.390**	0.004	19.859	0.448	63.251	1.168
FLAT_DRIP	0.545	0.010	19.800	0.327	102.330	1.217
LHT Env	0.654	0.010	**15.662**	0.363	33.657	0.416
Average	0.526	0.008	21.051	0.385	49.548	0.712

The best predictions for each trait in the five environments are in bold; the comparisons are made by column (trait). Traits are grain yield (GY), days to heading (DH), and plant height (PH)

## Discussion

The amounts of data that the breeding programs around the world are generating continue to increase; consequently, there is a growing need to extract more knowledge from the data being produced. To this end, multiple-trait models are commonly used to take advantage of correlated traits to improve parameter estimation and prediction accuracy. However, when there is a large number of traits, implementing these types of models is challenging. Therefore, it is necessary to develop efficient multiple-trait and multiple-environment models for whole-genome selection in order to take advantage of multiple correlated traits. In this paper, we propose an alternative method for analyzing multi-trait data that could be useful for whole-genome selection in the context of an abundance of traits. Some advantages of the proposed method are: (i) it can be implemented in current genomic selection software that was built for univariate analysis (e.g., BGLR, ASREML, package Sommer of R); (ii) it can be implemented with a large number of traits and, in general, with a large data set since this model is implemented in four steps; and (iii) this method can be more efficient in terms of implementation time because univariate analyses for each trait (step 2 of the procedure) are required for its implementation, which allows an implementation in parallel and with low dimensions compared to the BMTME model that models all the traits simultaneously and also (iv) decreases the probability of having collinearity and convergence problems, since when more correlated traits are added to the multivariate analysis, both problems increase (Schulthess et al. 2016).

In Supplementary material, we provide the R code for implementing the proposed method. When the matrix of response variable (***Y***) shows a considerable departure from normality, the proposed BMTME_Approx model is expected to be inefficient due to the fact that uncorrelated traits do not imply independence for non-normal data. For this reason, under these circumstances, in Supplementary material we also provide a solution to this situation based on the ICA that transforms the original unnecessary Gaussian matrix of response variables (***Y***) into a matrix of independent variables (Y^*^).

Also, it is important to point out that the BMTME_Thompson version is more efficient computationally than the original BMTME model since it allows sampling independently for each trait the random effects of ***b***_1_ and ***b***_2_, which improves computational efficiency because it avoids sampling from huge multivariate normal distributions. More specifically, the dimensions of the full conditionals are *L* times smaller than the original BMTME model. Another advantage is that the BMTME_Thompson model is not an approximation to the BMTME model, since it is only a reparametrization of the original model, which allows obtaining exactly the same parameter estimates and prediction accuracy at a lower cost in terms of implementation time, since the sampling process of the full conditionals of the random effects of ***b***_1_ and ***b***_2_ is done individually for each trait.

The proposed BMTME_Approx model takes advantage of the fact that optimal point estimates of any linear combination of the means and variances of the various separate analyses for each response variable can be obtained. Therefore, our proposed method uses a linear transformation of the separate parameter estimates to provide reasonable estimates of the beta coefficients (***β***), random effects (***b***_1_ and ***b***_2_), and variance–covariance matrices (**Σ**_*t*1_, **Σ**_*t*2_, and ***R***_*e*_) for a multivariate model. However, although the proposed model is able to provide reasonable approximate parameter estimates for the multivariate model by doing a separate analysis for each trait, it is unable to provide estimates of the standard error for off diagonal elements in the variance–covariance estimates.

Based on the results of the simulated and real data sets, we have reason to argue that the proposed BMTME_Approx model produces competitive predictions compared to those produced by the BMTME model, even though the parameter estimates resulting from the proposed BMTME_Approx model are quite different from those resulting from the BMTME model (mainly in the wheat real data set). However, the differences in parameter estimates between the BMTME and the BMTME_Approx models can be attributed to the fact that the BMTME_Approx model estimates two genetic variance–covariance matrices for traits (one for the interaction term genotype × trait and the other for the three-way interaction term environment × genotype × trait), while the BMTME only estimates one variance–covariance for both interaction terms. Also, in terms of prediction accuracies for simulated data set 1 (simulated with environments and correlated traits), the BMTME was better than the BMTME_Approx model, but in the second simulated data set, we observed that the BMTME_Approx (that assumes independence between environments and correlated traits) was better than the BMTME (that assumes correlated traits between environments and traits), which can be attributed to the fact that this second data set was simulated assuming independence between environments and correlated traits.

It is also important to point out that the proposed BMTME_Approx model can be implemented when there are many traits. For example, in the HTP and Large EYT data sets where there are a large number of traits, the application of the BMTME model becomes almost impossible due to the fact that samples are extracted from a very large number of multivariate normal distributions, and the modeling process is performed jointly for all the traits and not separately for each trait, as in the proposed BMTME_Approx model. This is a key element for achieving an efficient estimation process in terms of implementation time. Additionally, the proposed BMTME_Approx model can be implemented simultaneously since the procedure for estimating the required parameters requires a separate analysis for each trait.

Another point that we would like to highlight is that our proposed model is multiple-trait and multiple-environment but with the restriction that an identity matrix is assumed for the variance–covariance matrix of environments. However, even with this restrictive assumption in the variance–covariance matrix of environments, the model has the advantage of taking into account the interaction terms environment × trait, genotype × trait, and the three-way interaction environment × genotype × trait. Furthermore, it takes into account the correlated traits and can be implemented using conventional software for whole-genome prediction.

## Conclusions

The results of the simulated and real data sets show that the proposed alternative method produced results that are similar to those of the conventional multiple-trait analysis. For this reason, the proposed method is an attractive alternative for analyzing multiple-trait data in the context of a large number of traits. However, it is important to point out that the significant differences found in parameter estimates between the proposed BMTME_Approx model and the BMTME model can be attributed mainly to the fact that the BMTME_Approx model allows the estimation of two genetic variance–covariance matrices for traits, one for the interaction term genotype × trait, and the other for the term environment × genotype × trait, while the BMTME model only estimates one genetic matrix of variance–covariance for traits.

## Electronic supplementary material

SUPPLEMENTARY MATERIAL
