# Supplementary material for: A singular value decomposition Bayesian multiple-trait and multiple-environment genomic model
Source: Heredity (Edinb). 2018 Aug 17;122(4):381–401. doi: 10.1038/s41437-018-0109-7 (PMC6460759; doi:10.1038/s41437-018-0109-7)
Supplement: Supplementary file 1 — SUPPLEMENTARY MATERIAL [file 41437_2018_109_MOESM1_ESM.docx]

**SUPPLEMENTARY MATERIAL**

**Full conditionals of the BMTME model**

**Full conditional distribution for** $vec(\boldsymbol{\beta)}|$

$$P(vec(\boldsymbol{\beta)}|ELSE)\propto\exp\left\{ -\frac{1}{2}tr\left[ \boldsymbol{R}_{e}^{-1}\left( \boldsymbol{Y}-\boldsymbol{X\beta}-\boldsymbol{Z}_{1}\boldsymbol{b}_{1}-\boldsymbol{Z}_{2}\boldsymbol{b}_{2} \right)^{T}\boldsymbol{I}_{n}\left( \boldsymbol{Y}-\boldsymbol{X\beta}-\boldsymbol{Z}_{1}\boldsymbol{b}_{1}-\boldsymbol{Z}_{2}\boldsymbol{b}_{2} \right) \right]-\frac{1}{2}tr\left[ I_{L}^{-1}\left( \boldsymbol{\beta}-\boldsymbol{\beta}_{0} \right)^{T} \mathbf{I}_{I}^{-1}\left( \boldsymbol{\beta}-\boldsymbol{\beta}_{0} \right) \right] \right\}$$

$\propto exp\{-\frac{1}{2}[{vec\left( \boldsymbol{\beta} \right)-vec(\tilde{\boldsymbol{\beta}})]}^{T}\tilde{\Sigma}_{\boldsymbol{\beta}}^{-1}[vec\left( \boldsymbol{\beta} \right)-vec(\tilde{\boldsymbol{\beta}}) ]\}$

$\propto N(vec(\tilde{\boldsymbol{\beta}}),\tilde{\Sigma}_{\boldsymbol{\beta}})$ (A1)

where $\tilde{\Sigma}_{\boldsymbol{\beta}}=\left[ \mathbf{I}_{L}^{-1}\otimes\mathbf{I}_{I}^{-1}+\boldsymbol{R}_{e}^{-1}\otimes\boldsymbol{X}^{T}\boldsymbol{X} \right]^{-1}$, $vec(\tilde{\boldsymbol{\beta}})=\tilde{\Sigma}_{\boldsymbol{\beta}}\left\{ \left( \mathbf{I}_{L}^{-1}\otimes\mathbf{I}_{I}^{-1} \right)vec\left( \boldsymbol{\beta}_{0} \right)+\left( \boldsymbol{R}_{e}^{-1}\otimes\boldsymbol{X}^{T} \right)\left[ vec\left( \boldsymbol{Y} \right)-vec(\boldsymbol{Z}_{1}\boldsymbol{b}_{1})-vec(\boldsymbol{Z}_{2}\boldsymbol{b}_{2}) \right] \right\}$.

In the simplification of some calculations, the following properties were involved: $tr\left( \boldsymbol{AB} \right)=vec\left( \boldsymbol{A}^{T} \right)^{T}vec\left( \boldsymbol{B} \right)=vec\left( \boldsymbol{B} \right)^{T}vec(\boldsymbol{A}^{T}),$ and$vec \left( \boldsymbol{AXB} \right)=\boldsymbol{(B}^{T}\otimes\boldsymbol{A})vec(\boldsymbol{X})$.

**Full conditional for** $vec\left( \boldsymbol{b}_{1} \right)$

$$P\left( vec\left( \boldsymbol{b}_{1} \right) | ELSE \right)\propto\exp\left\{ -\frac{1}{2}[{vec\left( \boldsymbol{Y} \right) -vec(\boldsymbol{X\beta})-\boldsymbol{(I}_{L}\otimes\boldsymbol{Z}_{1}\boldsymbol{)}vec(\boldsymbol{b}_{1})-vec(\boldsymbol{Z}_{2}\boldsymbol{b}_{2})]}^{T} \left[ \boldsymbol{R}_{e}^{-1}\otimes\boldsymbol{I}_{n} \right] \left[ vec\left( \boldsymbol{Y} \right) -vec(\boldsymbol{X\beta})-\boldsymbol{(I}_{L}\otimes\boldsymbol{Z}_{1}\boldsymbol{)}vec(\boldsymbol{b}_{1})-vec(\boldsymbol{Z}_{2}\boldsymbol{b}_{2}) \right]-\frac{1}{2}\left[ {vec\left( \boldsymbol{b}_{1} \right)}^{T}\left[ \boldsymbol{\Sigma}_{t}^{-1}\otimes\boldsymbol{G}_{g}^{\boldsymbol{-1}} \right] \right]vec\left( \boldsymbol{b}_{1} \right) \right\}$$

$\propto\exp\left\{ -\frac{1}{2}\left[ vec\left( \boldsymbol{b}_{1} \right)-vec\left( {\tilde{\boldsymbol{b}}}_{1} \right) \right]^{T}{\tilde{\boldsymbol{\Sigma}}}_{\boldsymbol{b}_{1}}^{-1}\left[ vec\left( \boldsymbol{b}_{1} \right)-vec\left( {\tilde{\boldsymbol{b}}}_{1} \right) \right] \right\}$

$\propto N(vec\left( {\tilde{\boldsymbol{b}}}_{1} \right), {\tilde{\boldsymbol{\Sigma}}}_{\boldsymbol{b}_{1}}\boldsymbol{)}$ (A2)

where ${\tilde{\boldsymbol{\Sigma}}}_{\boldsymbol{b}_{1}}=\left( \boldsymbol{\Sigma}_{t}^{-1}\otimes\boldsymbol{G}_{g}^{-1}+\boldsymbol{R}_{e}^{-1}\otimes\boldsymbol{Z}_{1}^{T}\boldsymbol{Z}_{1} \right)^{-1}$ and $vec\left( {\tilde{\boldsymbol{b}}}_{1} \right)={\tilde{\boldsymbol{\Sigma}}}_{\boldsymbol{b}_{1}}\left( \boldsymbol{R}_{e}^{-1}\otimes\boldsymbol{Z}_{1}^{T} \right)\left[ vec\left( \boldsymbol{Y} \right)-vec\left( \boldsymbol{X\beta} \right)-vec(\boldsymbol{Z}_{2}\boldsymbol{b}_{2}) \right].$

**Full conditional for** $vec\left( \boldsymbol{b}_{2} \right)$

$$P\left( vec\left( \boldsymbol{b}_{2} \right) | ELSE \right)\propto\exp\left\{ -\frac{1}{2}\left[ vec\left( \boldsymbol{Y} \right) -vec\left( \boldsymbol{X\beta} \right)-vec\left( \boldsymbol{Z}_{1}\boldsymbol{b}_{1} \right)-\boldsymbol{(I}_{L}\otimes\boldsymbol{Z}_{2}\boldsymbol{)}vec(\boldsymbol{b}_{2}) \right]^{T}\left( \boldsymbol{R}_{e}^{-1}\otimes\boldsymbol{I}_{n} \right)\left[ vec\left( \boldsymbol{Y} \right) -vec\left( \boldsymbol{X\beta} \right)-vec\left( \boldsymbol{Z}_{1}\boldsymbol{b}_{1} \right)-\boldsymbol{(I}_{L}\otimes\boldsymbol{Z}_{2}\boldsymbol{)}vec(\boldsymbol{b}_{2}) \right]-\frac{1}{2}{vec\left( \boldsymbol{b}_{2} \right)}^{T}\left( \boldsymbol{\Sigma}_{t}^{-1}\otimes\boldsymbol{\Sigma}_{E}^{-1}\otimes\boldsymbol{G}_{g}^{\boldsymbol{-}1} \right)vec\left( \boldsymbol{b}_{2} \right) \right\}$$

$\propto\exp\left\{ -\frac{1}{2}\left[ vec\left( \boldsymbol{b}_{2} \right)-vec\left( {\tilde{\boldsymbol{b}}}_{2} \right) \right]^{T}{\tilde{\boldsymbol{\Sigma}}}_{\boldsymbol{b}_{2}}^{-1}\left[ vec\left( \boldsymbol{b}_{2} \right)-vec\left( {\tilde{\boldsymbol{b}}}_{2} \right) \right] \right\}$

$\propto N(vec\left( {\tilde{\boldsymbol{b}}}_{2} \right), {\tilde{\boldsymbol{\Sigma}}}_{\boldsymbol{b}_{2}}\boldsymbol{)}$ (A3)

where ${\tilde{\boldsymbol{\Sigma}}}_{\boldsymbol{b}_{2}}=\left( \boldsymbol{\Sigma}_{t}^{-1}\otimes\boldsymbol{\Sigma}_{E}^{-1}\otimes\boldsymbol{G}_{g}^{-1}+\boldsymbol{R}_{e}^{-1}\otimes\boldsymbol{Z}_{2}^{T}\boldsymbol{Z}_{2} \right)^{-1}$ and $vec\left( {\tilde{\boldsymbol{b}}}_{2} \right)={\tilde{\boldsymbol{\Sigma}}}_{\boldsymbol{b}_{2}}\left( \boldsymbol{R}_{e}^{-1}\otimes\boldsymbol{Z}_{2}^{T} \right)\left\{ vec\left( \boldsymbol{Y} \right)-vec\left( \boldsymbol{X\beta} \right)-vec(\boldsymbol{Z}_{1}\boldsymbol{b}_{1}) \right\}$.

**Full conditional for** $\boldsymbol{\Sigma}_{t}$

$$P\left( \boldsymbol{\Sigma}_{t} | ELSE \right)\propto P\left( \boldsymbol{b}_{1} | \boldsymbol{\Sigma}_{t} \right)P\left( \boldsymbol{b}_{2} | \boldsymbol{\Sigma}_{t} \right)P(\boldsymbol{\Sigma}_{t})$$

$$\propto\left| \boldsymbol{\Sigma}_{t} \right|^{-\frac{J}{2}}\left| \boldsymbol{G}_{g} \right|^{-\frac{L}{2}}\exp\left\{ -\frac{1}{2}tr\left[ \boldsymbol{b}_{1}^{T}\boldsymbol{G}_{g}^{-1}\boldsymbol{b}_{1}\boldsymbol{\Sigma}_{t}^{-1} \right] \right\}$$

$$\times\left| \boldsymbol{\Sigma}_{t} \right|^{-\frac{IJ}{2}}\left| \boldsymbol{\Sigma}_{E}\otimes\boldsymbol{G}_{g} \right|^{-\frac{L}{2}}\exp\left\{ -\frac{1}{2}tr\left[ \boldsymbol{b}_{2}^{T}\left( \boldsymbol{\Sigma}_{E}^{-1}\otimes\boldsymbol{G}_{g}^{-1} \right)\boldsymbol{b}_{2}\boldsymbol{\Sigma}_{t}^{-1} \right] \right\}P(\boldsymbol{\Sigma}_{t})$$

$$\propto\boldsymbol{\Sigma}_{t}^{-\frac{\upsilon_{t}+J+IJ+L-1+1}{2}}\exp\left\{ -\frac{1}{2}tr\left( \boldsymbol{b}_{1}^{T}\boldsymbol{G}_{g}^{-1}\boldsymbol{b}_{1}+\boldsymbol{b}_{2}^{T}\left( \boldsymbol{\Sigma}_{E}^{-1}\otimes\boldsymbol{G}_{g}^{-1} \right)\boldsymbol{b}_{2}+\boldsymbol{S}_{t} \right)\boldsymbol{\Sigma}_{t}^{-1} \right\}$$

$\propto IW\left( \upsilon_{t}+J+L+IJ-1,\boldsymbol{b}_{1}^{T}\boldsymbol{G}_{g}^{-1}\boldsymbol{b}_{1}+\boldsymbol{b}_{2}^{T}\left( \boldsymbol{\Sigma}_{E}^{-1}\otimes\boldsymbol{G}_{g}^{-1} \right)\boldsymbol{b}_{2}+\boldsymbol{S}_{t} \right)$ (A4)

**Full conditional for** $\boldsymbol{\Sigma}_{E}$

$$P\left( \boldsymbol{\Sigma}_{E} | ELSE \right)\propto P(\boldsymbol{b}_{2}|\boldsymbol{\Sigma}_{E}) P(\boldsymbol{\Sigma}_{E})$$

$\propto\left| \boldsymbol{\Sigma}_{E} \right|^{-\frac{JL}{2}}\left| \boldsymbol{G}_{g}\otimes\boldsymbol{\Sigma}_{t} \right|^{-\frac{I}{2}}\exp\left\{ -\frac{1}{2}tr\left[ \boldsymbol{b}_{2}^{*T}\left( \boldsymbol{G}_{g}^{-1}\otimes\boldsymbol{\Sigma}_{t}^{-1} \right)\boldsymbol{b}_{2}^{*}\boldsymbol{\Sigma}_{E}^{-1} \right] \right\}\left| \boldsymbol{S}_{E} \right|^{\frac{\upsilon_{E}+I-1}{2}}\times\left| \boldsymbol{\Sigma}_{E} \right|^{-\frac{\upsilon_{E}+I}{2}}\exp\left\{ -\frac{1}{2}tr\left( \boldsymbol{S}_{E}\boldsymbol{\Sigma}_{E}^{-1} \right) \right\}$

${\propto\left| \boldsymbol{\Sigma}_{E} \right|}^{-\frac{\upsilon_{E}+I+JL}{2}}\exp\left\{ -\frac{1}{2}tr\left[ \boldsymbol{(b}_{2}^{*T}\left( \boldsymbol{G}_{g}^{-1}\otimes\boldsymbol{\Sigma}_{t}^{-1} \right)\boldsymbol{b}_{2}^{*}+\boldsymbol{S}_{E}) \right]\boldsymbol{\Sigma}_{E}^{-1} \right\}\left| \boldsymbol{S}_{E} \right|^{\frac{\upsilon_{E}+I-1}{2}}$

$\propto IW(\upsilon_{E}+JL+I-1, \boldsymbol{b}_{2}^{*T}\left( \boldsymbol{G}_{g}^{-1}\otimes\boldsymbol{\Sigma}_{t}^{-1} \right)\boldsymbol{b}_{2}^{*}+\boldsymbol{S}_{E})$ (A5)

**Full conditional for** $\mathbf{R}_{e}$

$$P\left( \mathbf{R}_{e} | ELSE \right)\propto P\left( \boldsymbol{Y} | \boldsymbol{\beta},\boldsymbol{b}_{1},\boldsymbol{b}_{2},\mathbf{R}_{e} \right)P\left( \mathbf{R}_{e} \right)$$

$$\propto\left| \mathbf{R}_{e} \right|^{-\frac{n}{2}}\exp\left\{ -\frac{1}{2}tr\left[ \left\| \left( \boldsymbol{Y}-\boldsymbol{X\beta}-\boldsymbol{Z}_{1}\boldsymbol{b}_{1}-\boldsymbol{Z}_{2}\boldsymbol{b}_{2} \right) \right\|\boldsymbol{R}_{e}^{-1} \right] \right\}\times\left| \boldsymbol{S}_{e} \right|^{\frac{\upsilon_{e}+L-1}{2}}\left| \mathbf{R}_{e} \right|^{-\frac{\upsilon_{e}+L}{2}}\exp\left\{ -\frac{1}{2}tr(\boldsymbol{S}_{e}\boldsymbol{R}_{e}^{-1}) \right\}$$

$\propto\left| \mathbf{R}_{e} \right|^{-\frac{\upsilon_{e}+n+L-1+1}{2}}\exp\left\{ -\frac{1}{2}tr\left[ \left\| \left( \boldsymbol{Y}-\boldsymbol{X\beta}-\boldsymbol{Z}_{1}\boldsymbol{b}_{1}-\boldsymbol{Z}_{2}\boldsymbol{b}_{2} \right) \right\|+\boldsymbol{S}_{e} \right]\boldsymbol{R}_{e}^{-1} \right\}$

$\propto IW\left( \upsilon_{e}+n+L-1,\left\| \left( \boldsymbol{Y}-\boldsymbol{X\beta}-\boldsymbol{Z}_{1}\boldsymbol{b}_{1}-\boldsymbol{Z}_{2}\boldsymbol{b}_{2} \right) \right\|+\boldsymbol{S}_{e} \right)$ (A6)

where ${\left\| \left( \boldsymbol{Y}-\boldsymbol{X\beta}-\boldsymbol{Z}_{1}\boldsymbol{b}_{1}-\boldsymbol{Z}_{2}\boldsymbol{b}_{2} \right) \right\|=\left( \boldsymbol{Y}-\boldsymbol{X\beta}-\boldsymbol{Z}_{1}\boldsymbol{b}_{1}-\boldsymbol{Z}_{2}\boldsymbol{b}_{2} \right)}^{T}\left( \boldsymbol{Y}-\boldsymbol{X\beta}-\boldsymbol{Z}_{1}\boldsymbol{b}_{1}-\boldsymbol{Z}_{2}\boldsymbol{b}_{2} \right)$.

**Setting the hyper-parameters for the prior distributions of the BMTME model**

The hyper-parameters for the BMTME model were set similar to those used in the BGLR software (Pérez-Rodríguez and de los Campos, 2014). These rules provide proper but weakly informative prior distributions so that we partitioned the total variance-covariance of the phenotypes into two components: (1) the error and (2) the linear predictor. First we provide the variance-covariance of the phenotypes

$Var(vec\left( \boldsymbol{Y} \right))=\boldsymbol{\Sigma}_{\beta t}\otimes\boldsymbol{X}\boldsymbol{X}^{T}$**+**$\boldsymbol{\Sigma}_{t}\otimes\boldsymbol{Z}_{\boldsymbol{1}}{\boldsymbol{G}_{g}\boldsymbol{Z}}_{\boldsymbol{1}}^{\boldsymbol{T}}$**+**$\boldsymbol{\Sigma}_{t}\otimes\boldsymbol{Z}_{\boldsymbol{2}}{{\boldsymbol{(}\boldsymbol{\Sigma}_{E}\otimes\boldsymbol{G}}_{g}\boldsymbol{)Z}}_{\boldsymbol{2}}^{\boldsymbol{T}}$**+**$\mathbf{R}_{e}\otimes\boldsymbol{I}_{n}$ (C1)

Therefore, the variance-covariance of row $i$ for $i=1,2,\ldots,n$ of $\boldsymbol{Y}$ is equal to

${Var(\boldsymbol{y}}_{i})=\boldsymbol{\Sigma}_{\beta t}\boldsymbol{x}_{i}^{T}\boldsymbol{x}_{i}$**+**$\boldsymbol{\Sigma}_{t}\boldsymbol{z}_{1i}^{T}{\boldsymbol{G}_{g}\boldsymbol{z}}_{1i}$**+**$\boldsymbol{\Sigma}_{t}\boldsymbol{z}_{2i}^{T}{{\boldsymbol{(}\boldsymbol{\Sigma}_{E}\otimes\boldsymbol{G}}_{g}\boldsymbol{)}\boldsymbol{z}}_{2i}$**+**$\mathbf{R}_{e}$ (C2)

Therefore, the average of the $n$ rows of equation (C2), called total variance, is equal to

${Var(\bar{\boldsymbol{y}}}_{i})=\boldsymbol{\Sigma}_{\beta t}\sum_{i=1}^{n} \boldsymbol{x}_{i}^{T}\boldsymbol{x}_{i}/n$**+**$\boldsymbol{\Sigma}_{t}(\sum_{i=1}^{n} \boldsymbol{z}_{1i}^{T}{\boldsymbol{G}_{g}\boldsymbol{z}}_{1i})/n$**+**$\boldsymbol{\Sigma}_{t}(\sum_{i=1}^{n} \boldsymbol{z}_{2i}^{T}{{\boldsymbol{(}\boldsymbol{\Sigma}_{E}\otimes\boldsymbol{G}}_{g}\boldsymbol{)}\boldsymbol{z}}_{2i})/n$**+**$\mathbf{R}_{e}$

${Var(\bar{\boldsymbol{y}}}_{i})=\boldsymbol{\Sigma}_{\beta t}{MS}_{\beta t}$**+**$\boldsymbol{\Sigma}_{t}{MS}_{b1}$**+**${\boldsymbol{\Sigma}_{t}MS}_{b2}$**+**$\mathbf{R}_{e}$

${Var(\bar{\boldsymbol{y}}}_{i})=\mathbf{V}_{y}=\mathbf{V}_{\beta t}$+$\mathbf{V}_{b1}+\mathbf{V}_{b2}+\mathbf{R}_{e}$ (C3)

where: ${MS}_{\beta t}=\sum_{i=1}^{n} \boldsymbol{x}_{i}^{T}\boldsymbol{x}_{i}/n$, ${MS}_{b1}=(\sum_{i=1}^{n} \boldsymbol{z}_{1i}^{T}{\boldsymbol{G}_{g}\boldsymbol{z}}_{1i})/n$, ${MS}_{b2}=(\sum_{i=1}^{n} \boldsymbol{z}_{2i}^{T}{{\boldsymbol{(}\boldsymbol{\Sigma}_{E}\otimes\boldsymbol{G}}_{g}\boldsymbol{)}\boldsymbol{z}}_{2i})/n$, and

$\mathbf{V}_{\beta t}=\boldsymbol{\Sigma}_{\beta t}{MS}_{\beta t}$ (C4)

$\mathbf{V}_{b1}=\boldsymbol{\Sigma}_{t}{MS}_{b1}$ (C5)

$\mathbf{V}_{b2}=\boldsymbol{\Sigma}_{t}{MS}_{b2}$ (C6)

*Setting the hyper-parameters for* $\boldsymbol{\Sigma}_{\beta t}$

Since $E(\boldsymbol{\Sigma}_{\beta t}\left| {df}_{\beta t}, \right.\mathbf{S}_{\beta t}$)=$\frac{\mathbf{S}_{\beta t}}{{df}_{\beta t}-L-1}$ and mode$\left( \boldsymbol{\Sigma}_{\beta t}\left| {df}_{\beta t}, \right.\mathbf{S}_{\beta t} \right)=\frac{\mathbf{S}_{\beta t}}{{df}_{\beta t}+L+1}$, for ${df}_{\beta t}>L+1$. Therefore, from equation (C4),

$\boldsymbol{\Sigma}_{\beta t}=\mathbf{V}_{\beta t}/{MS}_{\beta t}$ (C7)

Thus if we replace the left-hand side of equation (C7) with the mode of $\boldsymbol{\Sigma}_{\beta t}$, then

$\frac{\mathbf{S}_{\beta t}}{{df}_{\beta t}+L+1}=\frac{\mathbf{V}_{\beta t}}{{MS}_{\beta t}}$ (C8)

From (C8) we get $\mathbf{S}_{\beta t}=\frac{\mathbf{V}_{\beta t}\times{(df}_{\beta t}+L+1)}{{MS}_{\beta t}}$. Then by setting $R_{1}^{2}$ as the proportion of the total variance-covariance ($\mathbf{V}_{y}$) that a priori is explained by the traits, $\mathbf{V}_{\beta t}=R_{1}^{2}\mathbf{V}_{y}$, we have that

$\mathbf{S}_{\beta t}$=$\frac{R_{1}^{2}\mathbf{V}_{y}\times{(df}_{\beta t}+L+1)}{{MS}_{\beta t}}$ (C9)

Once we set ${df}_{\beta t}$, we can set $\mathbf{S}_{\beta t}$ as in (C9) and we only need to compute the phenotypic variance-covariance matrix ($\mathbf{V}_{y}$), ${MS}_{\beta t}$ and set $R_{1}^{2}$ as the proportion of variance-covariance that a priori is explained by the traits. We set $R_{1}^{2}=0.25$ as default.

*Setting the hyper-parameters for* $\boldsymbol{\Sigma}_{t}$

Also, since $E(\boldsymbol{\Sigma}_{t}\left| {df}_{t1}, \right.\mathbf{S}_{t1}$)=$\frac{\mathbf{S}_{t1}}{{df}_{t1}-L-1}$ and mode$\left( \boldsymbol{\Sigma}_{t}\left| {df}_{t1}, \right.\mathbf{S}_{t1} \right)=\frac{\mathbf{S}_{t1}}{{df}_{t1}+L+1}$, for ${df}_{t1}>L+$ , and using equations (C5) and (C6) in similar way as before,

$\boldsymbol{\Sigma}_{t}=\frac{\mathbf{V}_{b1}}{{MS}_{b1}}+\frac{\mathbf{V}_{b2}}{{MS}_{b2}}$ (C10)

Thus if we replace the left-hand side of equation (C9) with the mode of $\boldsymbol{\Sigma}_{t}$, then

$\frac{\mathbf{S}_{t}}{{df}_{t}+L+1}=\frac{\mathbf{V}_{b1}}{{MS}_{b1}}+\frac{\mathbf{V}_{b2}}{{MS}_{b2}}$ (C11)

From (C11) and solving for $\mathbf{S}_{t}=\frac{\mathbf{V}_{b1}\times{(df}_{t}+L+1)}{{MS}_{b1}}+\frac{\mathbf{V}_{b2}\times{(df}_{t}+L+1)}{{MS}_{b2}}$, then

$\mathbf{S}_{t}=\frac{R_{2}^{2}\mathbf{V}_{y}\times{(df}_{t}+L+1)}{{MS}_{b1}}+\frac{R_{3}^{2}\mathbf{V}_{y}\times{(df}_{t}+L+1)}{{MS}_{b2}}$ (C12)

for which we only need to compute the phenotypic variance-covariance matrix ($\mathbf{V}_{y}$), ${MS}_{b1},$ ${MS}_{b2}$ and set $R_{2}^{2}$ and $R_{3}^{2}$ as the proportion of variance-covariance that a priori is explained by the traits in the interaction terms genotype$\times$trait and genotype$\times$environment$\times$trait. We set $R_{2}^{2}=R_{3}^{2}=0.25$ as default. In the balanced case, ${MS}_{b2}=tr\left( \boldsymbol{Z}_{2}\left( \boldsymbol{\Sigma}_{E}\otimes\boldsymbol{G} \right)\boldsymbol{Z}_{2}^{T} \right)=tr\left( \boldsymbol{Z}_{2}^{T}\boldsymbol{Z}_{2}\left( \boldsymbol{\Sigma}_{E}\otimes\boldsymbol{G} \right) \right)=tr\left( \boldsymbol{\Sigma}_{E}\otimes\boldsymbol{G} \right)=tr\left( \mathbf{G} \right)tr\left( \boldsymbol{\Sigma}_{E} \right)$, so to complete the setting value of ${MS}_{b2}$, we take $tr\left( \boldsymbol{\Sigma}_{E} \right)=\frac{1}{tr\left( G \right)}\frac{1}{3L}\sum_{l=1}^{L} V_{\boldsymbol{y}_{l}}$, where $V_{\boldsymbol{y}_{l}}$ is the phenotypic variance of trait $l$.

*Setting the hyper-parameters for* $\boldsymbol{\Sigma}_{E}$

Also, since $E(\boldsymbol{\Sigma}_{E}\left| {df}_{E}, \right.\mathbf{S}_{E}$)=$\frac{\mathbf{S}_{E}}{{df}_{E}-I-1}$ and mode$\left( \boldsymbol{\Sigma}_{E}\left| {df}_{E}, \right.\mathbf{S}_{E} \right)=\frac{\mathbf{S}_{E}}{{df}_{E}+I+1}$, for ${df}_{E}>I+1$. Let$\mathbf{V}_{y*}$ be the variance-covariance matrix of the matrix of phenotypic responses, $\boldsymbol{Y}^{*}$, that resulted, but accommodating the information of the matrix of phenotypic responses ($\boldsymbol{Y}$) of order $n\times L$, with $n$ =IJ, as a matrix of order $n^{*}\times I$, with $n^{*}=JL$, that is, the columns of $\boldsymbol{Y}^{*}$ correspond to environments instead of traits as in $\boldsymbol{Y}$. Then in similar fashion, we can define

$\boldsymbol{\Sigma}_{E}=\mathbf{V}_{b2*}/{MS}_{b2*}$ (C13)

Thus, if we replace the left-hand side of equation (C13) with the mode of $\boldsymbol{\Sigma}_{E}$, then

$\frac{\mathbf{S}_{E}}{{df}_{E}+L+1}=\frac{\mathbf{V}_{b2*}}{{MS}_{b2*}}$ (C14)

From (C14) and solving for $\mathbf{S}_{E}=\frac{\mathbf{V}_{b2*}\times{(df}_{E}+L+1)}{{MS}_{b2}}$, then

$\mathbf{S}_{E}=\frac{R_{3}^{2}\mathbf{V}_{y*}\times{(df}_{E}+I+1)}{{MS}_{b2*}}$ (C15)

With ${MS}_{b2*}=tr\left( \frac{\mathbf{S}_{t}}{{df}_{t}+L+1}\bigotimes\boldsymbol{G} \right)$

*Setting the hyper-parameters for* $\mathbf{R}_{e}$

Also, since $E(\mathbf{R}_{e}\left| {df}_{e}, \right.\mathbf{S}_{e}$)=$\frac{\mathbf{S}_{e}}{{df}_{e}-L-1}$ and mode$\left( \mathbf{R}_{e}\left| {df}_{e}, \right.\mathbf{S}_{e} \right)=\frac{\mathbf{S}_{e}}{{df}_{e}+L+1}$, for ${df}_{e}>L+1$. Therefore, in similar fashion to the above hyper-parameters, we set

$\mathbf{S}_{e}={(1-R_{1}^{2}-R}_{2}^{2}-R_{3}^{2})\mathbf{V}_{y}\times{(df}_{e}+L+1)$ (C16)

**R code for the implementation of the proposed BMTME_Approx model under SVD**

#######################################################################

rm(list=objects()); ls()

setwd("C:\\TELEMATICA 2017\\Fernando Teledo\\FA MODEL")

library(BGLR)

############Iterations, burning and thining#################

nIter=2000

burnIn=1000

thin=10

###############Loading the Genomic relationship matrix#################

load('Gg.RData')

Hybrids=data.frame(read.table('DHHybridsNewRun.Run.csv',sep=',',h=T));

Hybrids$PH=Hybrids$PH/100

Hybrids=Hybrids[order(Hybrids$Env, Hybrids$Line), ]

Y.ave.ord=data.frame(Hybrids[,-1])

y=Y.ave.ord[,c(3:5)]

#####Cholesly decomposition##########################################

K <-Gg

LL=t(chol(Gg))

Z11=model.matrix(~0+factor(Y.ave.ord$Line))

Z1=Z11%*%LL

Z2=model.matrix(~0+Z1:factor(Y.ave.ord$Env))

X=model.matrix(~0+factor(Y.ave.ord$Env))

##precalculations

y=data.matrix(Y.ave.ord[,c(3:5)])

y2=y

nt=ncol(y2) ##################Number of traits########################

nI=ncol(X) ##################Number of environments###################

nJ=length(unique(Y.ave.ord$Line)) ##################Number of genotypes

ri=1 #################Number of replications##########################

rownames(y2)=c(1:(nI*nJ))

y1=y2

Y=y1

#################Singular value decomposition###########################

SVD_Y=svd(Y)

U=SVD_Y$u

V=SVD_Y$v

tV=t(V)

K_L=Z1%*%t(Z1)

K_LE=Z2%*%t(Z2)

################Transformed response variable######################

Ytilde=Y%*%V

dim(Ytilde)

Y_pred=matrix(NA,nrow=nrow(Y),ncol=ncol(Y))

Beta_PC=matrix(NA,nrow=ncol(X),ncol=ncol(Y))

SDBeta_PC=matrix(NA,nrow=ncol(X),ncol=ncol(Y))

Sigma1_PC=matrix(0,nrow=ncol(Y),ncol=ncol(Y))

Sigma2_PC=matrix(0,nrow=ncol(Y),ncol=ncol(Y))

SigmaError_PC=matrix(0,nrow=ncol(Y),ncol=ncol(Y))

SD1_PC=matrix(0,nrow=ncol(Y),ncol=ncol(Y))

SD2_PC=matrix(0,nrow=ncol(Y),ncol=ncol(Y))

SDError_PC=matrix(0,nrow=ncol(Y),ncol=ncol(Y))

for (i in 1:nt){

y2=Ytilde[,i]

ETA1=list(Env=list(X=X[,-3],model="FIXED"),Gen=list(K=K_L,model="RKHS"), GenxEnv=list(K=K_LE,model="RKHS"))

fm1=BGLR(y=y2,ETA=ETA1, nIter=nIter,burnIn=burnIn)

yhat2=fm1$yHat

betas_est=fm1$mu+fm1$ETA[[1]]$b

SDbetas_est=fm1$SD.mu+fm1$ETA[[1]]$SD.b

Y_pred[,i]=yhat2

Beta_PC[,i]=c(betas_est,fm1$mu)

SDBeta_PC[,i]=c(SDbetas_est,fm1$SD.mu)

Sigma1_PC[i,i]=fm1$ETA[[2]]$varU

SD1_PC[i,i]=fm1$ETA[[2]]$SD.varU

Sigma2_PC[i,i]=fm1$ETA[[3]]$varU

SD2_PC[i,i]=fm1$ETA[[3]]$SD.varU

SigmaError_PC[i,i]=fm1$varE

SDError_PC[i,i]=fm1$SD.varE

}

Y_pred_Final=Y_pred%*%tV

Beta_Orig=Beta_PC%*%tV

SDBeta_Orig=SDBeta_PC%*%tV

Sigma_T=V%*%Sigma1_PC%*%tV

SD_T=SD1_PC%*%tV

Sigma_T2=V%*%Sigma2_PC%*%tV

SD_T2=SD2_PC%*%tV

Sigma_Error=V%*%SigmaError_PC%*%tV

SD_Error=SDError_PC%*%tV

#########Parameter estimates and predicted values###############

Beta_Orig

Sigma_T

Sigma_T2

Sigma_Error

cov2cor(Sigma_T)

cov2cor(Sigma_T2)

cov2cor(Sigma_Error)

colnames(Y_pred_Final)=colnames(Y)

cbind(Y,Y_pred_Final)

**INDEPENDENT COMPONENT ANALYSES (ICA)**

Here, we provide a version of the proposed model based on independent component analysis

(ICA) that transforms the distribution of the subcomponents of original unnecessary Gaussian

matrix of correlated response variables ($\boldsymbol{Y)}$ into a matrix with subcomponents independent

($\boldsymbol{Y}^{\boldsymbol{*}}\boldsymbol{)}$. The proposed method given in this paper in equations (3-10) and that consist of four

steps is appropriate for normally distributed traits since in the context of normality, two

uncorrelated traits imply two independent traits. However, when there is considerable

departure from normality, the assumption of independence of the subcomponents between

any two traits is weak or null when the traits are uncorrelated but are weakly or strongly when

the trait are far away from the assumption of normality. In these circumstances, the proposed

BMTME_Approx model is expected to be inefficient for the approximation of the BMTME,

since even the traits that are uncorrelated will not be independent due to the departure from

normality of some or all traits. This problem can be solved by using independent component

analysis (ICA). The ICA transforms the original matrix of response variable ($\boldsymbol{Y)}$ that its

subcomponents are assumed non-normally distributed into a set of independent (or as

independent as possible) response variables. This is possible because ICA is a computational

method that decomposes a multivariate signal into independent non-Gaussian signals, which

is done by assuming that the subcomponents are non-Gaussian signals and statistically

independent of each other (Hyvärinen et al., 2001; Stone, 2004). However, to avoid asking

about the minimum number of traits that need to be normally distributed to apply the SVD

method described above and transform the correlated traits into uncorrelated ones, we suggest

verifying the normality assumption jointly for all the traits (using any test of normality for

multivariate data) under study; if there is a significant statistical departure from a multivariate

normal distribution of the traits in this situation, we suggest implementing the ICA to

transform the original response variables into independent traits that are non-normally

distributed. However, if there is a strong departure from normality, even when the application

of the ICA guarantee independence of the transformed traits, a perfect performance by the

proposed model is not guaranteed, as our model was built under the assumption that the traits

are multivariate normal distributed.

In ICA, it is assumed that the matrix of responses is $\boldsymbol{Y=}\boldsymbol{SA}$, where $\boldsymbol{S}$ is the matrix of the hidden components that are assumed independent. The task is to transform the distribution of the subcomponents of observed data ($\boldsymbol{Y)}$ {\displaystyle {\boldsymbol {x}},}using a linear transformation {\displaystyle {\boldsymbol {W}}}$\boldsymbol{A}^{\boldsymbol{-1}}$ as $\boldsymbol{S=}${\displaystyle {\boldsymbol {s}}={\boldsymbol {W}}{\boldsymbol {x}},} $\boldsymbol{Y}\boldsymbol{A}^{\boldsymbol{-1}}$ into an observable vector that its subcomponents are of independent or as much as independent,{\displaystyle {\boldsymbol {s}}} $\boldsymbol{Y}^{*}=\boldsymbol{S}=\boldsymbol{Y}\boldsymbol{A}^{\boldsymbol{-1}}$ (Hyvärinen et al., 2001). The ICA can be implemented in R with the FastICA library. Therefore, BMTME_Approx in terms of ICA can be implemented using the R code given below.

Implementation with ICA is exactly the same as the implementation described in the four steps above, with the only difference being that step 1 has been modified, since the de-correlation process is performed with the ICA algorithm, assuming $\boldsymbol{Y=}\boldsymbol{SA}$ and is used as response variable $\boldsymbol{Y}^{*}=\boldsymbol{Y}\boldsymbol{A}^{\boldsymbol{-1}}$. To recover the predicted values and parameter estimates in equations (4-10), we replaced $\boldsymbol{V}^{T}$ with $\boldsymbol{A}$. As stated above, implementation of the proposed model was done using the R package (2018).

**R code for the implementation of the proposed BMTME_Approx model under ICA**

rm(list=objects()); ls()

setwd("C:\\TELEMATICA 2017\\Fernando Teledo\\FA MODEL")

library(BGLR)

library(fastICA)

############Iterations, burning and thining#################

nIter=2000

burnIn=1000

thin=10

###############Loading the Genomic relationship matrix#################

load('Gg.RData')

Hybrids=data.frame(read.table('DHHybridsNewRun.Run.csv',sep=',',h=T));

Hybrids$PH=Hybrids$PH/100

Hybrids=Hybrids[order(Hybrids$Env, Hybrids$Line), ]

Y.ave.ord=data.frame(Hybrids[,-1])

y=Y.ave.ord[,c(3:5)]

#####Cholesly decomposition##########################################

K <-Gg

LL=t(chol(Gg))

Z11=model.matrix(~0+factor(Y.ave.ord$Line))

Z1=Z11%*%LL

Z2=model.matrix(~0+Z1:factor(Y.ave.ord$Env))

X=model.matrix(~0+factor(Y.ave.ord$Env))

##precalculations

y=data.matrix(Y.ave.ord[,c(3:5)])

y2=y

nt=ncol(y2) ##################Number of traits########################

nI=ncol(X) ##################Number of environments###################

nJ=length(unique(Y.ave.ord$Line)) ##################Number of genotypes

ri=1 #################Number of replications##########################

rownames(y2)=c(1:(nI*nJ))

y1=y2

Y=y1

SVD_ICA=fastICA(Y,3,alg.typ = "parallel", fun = "logcosh", alpha = 1,method = "C", row.norm=FALSE, maxit = 200,tol = 0.0001, verbose = TRUE)

K_L=Z1%*%t(Z1)

K_LE=Z2%*%t(Z2)

A=SVD_ICA$A

W2=solve(A)

Ytilde=Y%*%W2

dim(Ytilde)

Y_pred=matrix(NA,nrow=nrow(Y),ncol=ncol(Y))

Beta_PC=matrix(NA,nrow=ncol(X),ncol=ncol(Y))

SDBeta_PC=matrix(NA,nrow=ncol(X),ncol=ncol(Y))

Sigma1_PC=matrix(0,nrow=ncol(Y),ncol=ncol(Y))

Sigma2_PC=matrix(0,nrow=ncol(Y),ncol=ncol(Y))

SigmaError_PC=matrix(0,nrow=ncol(Y),ncol=ncol(Y))

SD1_PC=matrix(0,nrow=ncol(Y),ncol=ncol(Y))

SD2_PC=matrix(0,nrow=ncol(Y),ncol=ncol(Y))

SDError_PC=matrix(0,nrow=ncol(Y),ncol=ncol(Y))

for (i in 1:3){

y2=Ytilde[,i]

ETA1=list(Env=list(X=X[,-3],model="FIXED"),Gen=list(K=K_L,model="RKHS"), GenxEnv=list(K=K_LE,model="RKHS"))

fm1=BGLR(y=y2,ETA=ETA1, nIter=nIter,burnIn=burnIn)

yhat2=fm1$yHat

betas_est=fm1$mu+fm1$ETA[[1]]$b

SDbetas_est=fm1$SD.mu+fm1$ETA[[1]]$SD.b

Y_pred[,i]=yhat2

Beta_PC[,i]=c(betas_est,fm1$mu)

SDBeta_PC[,i]=c(SDbetas_est,fm1$SD.mu)

Sigma1_PC[i,i]=fm1$ETA[[2]]$varU

SD1_PC[i,i]=fm1$ETA[[2]]$SD.varU

Sigma2_PC[i,i]=fm1$ETA[[3]]$varU

SD2_PC[i,i]=fm1$ETA[[3]]$SD.varU

SigmaError_PC[i,i]=fm1$varE

SDError_PC[i,i]=fm1$SD.varE

}

Y_pred_Final=Y_pred%*%A

Beta_Orig=Beta_PC%*%A

SDBeta_Orig=SDBeta_PC%*%A

Sigma_T=t(A)%*%Sigma1_PC%*%A

SD_T=SD1_PC%*%A

Sigma_T2=t(A)%*%Sigma2_PC%*%A

SD_T2=SD2_PC%*%A

Sigma_Error=t(A)%*%SigmaError_PC%*%A

SD_Error=SDError_PC%*%A

Beta_Orig

Sigma_T

Sigma_T2

cov2cor(Sigma_T)

cov2cor(Sigma_T2)

cov2cor(Sigma_Error)

colnames(Y_pred_Final)=colnames(Y)

cbind(Y,Y_pred_Final)
